# Activation of FANCC attenuates mitochondrial ROS-driven necroptosis by targeting TBK1-dependent mitophagy in astrocytes after spinal cord injury

**DOI:** 10.7150/thno.109071

**Published:** 2025-03-18

**Authors:** Mingjie Xia, Chaochen Li, Jiajia Chen, Chunshuai Wu, Jinlong Zhang, Hongxiang Hong, Jiawei Jiang, Guanhua Xu, Zhanyang Qian, Zhiming Cui

**Affiliations:** 1 Department of Spine Surgery, The Second Affiliated Hospital of Nantong University, Nantong First People's Hospital, Medical School of Nantong University, Nantong, Jiangsu 226000, China.; 2 Research Institute for Spine and Spinal Cord Disease of Nantong University, Nantong, Jiangsu 226000, China.; 3 Key Laboratory of Neuroregeneration of Jiangsu and Ministry of Education, Nantong University, Nantong, Jiangsu 226000, China.

**Keywords:** FANCC, Necroptosis, Mitophagy, TBK1, Spinal cord injury

## Abstract

**Rationale:** Necroptosis in astrocytes induced by mitochondrial dysfunction following spinal cord injury (SCI) significantly contributes to neuronal functional deficits. Mitophagy plays a crucial role in clearing damaged mitochondria and inhibiting necroptosis. Fanconi anemia complementation group C (FANCC), a member of the Fanconi anemia gene family, exerts a protective role by facilitating mitophagy in immune processes. However, the role of FANCC in SCI-induced astrocytic necroptosis and the underlying mechanisms remain unexplored.

**Methods:** Astrocyte-specific FANCC conditional knockout (*Fancc^fl/fl^-GFAP-Cre*) mice, obtained by mating *Fancc^fl/fl^* mice with *GFAP-Cre* mice, served as a model of moderate thoracic spinal cord contusion injuries. Using bulk and single-nucleus RNA sequencing, we investigated the protective role of FANCC in astrocytes after SCI. We assessed necroptosis and mitophagy in astrocytes through quantitative PCR, western blotting, flow cytometry, immunofluorescence, and transmission electron microscopy. Molecular mechanisms were explored via co-immunoprecipitation, proteomics, molecular docking, and confocal imaging. Computer virtual screening identified poliumoside as a FANCC activator. Histopathological staining and functional assessments (gait analysis, Basso Mouse Scale, and hindlimb reflex score) were conducted to evaluate the therapeutic effects of poliumoside on SCI.

**Results:** Astrocytic FANCC deficiency exacerbated necroptosis and mitochondrial damage, leading to severe neurological deficits. Conversely, FANCC overexpression increased PTEN-induced kinase 1-Parkin expression, thereby activating mitophagy and reducing necroptosis. Proteomics revealed FANCC's interaction with a specific peptide of TANK-binding kinase 1 (TBK1), which further promoted mitophagy. Treatment with the FANCC activator poliumoside improved neural pathology and motor function recovery in SCI mice.

**Conclusion:** The current study indicated that FANCC interacts with TBK1 and consequently mediates Parkin translocation, activates mitophagy, and inhibits astrocyte necroptosis. Our findings demonstrate the neuroprotective role and therapeutic potential of FANCC for SCI amelioration.

## Introduction

Spinal cord injury (SCI) is a neurological disorder that is caused by traumatic events, such as high falls and traffic accidents, with an overall global incidence of ~130 per million 1. Approximately 2-3 million people worldwide are disabled due to SCI 2,3. Patients suffering from paraplegia or quadriplegia with bladder, bowel, or sexual dysfunction experience a significant decline in their quality of life and represent a heavy burden on their families and society 4,5. The current treatment options for SCI are limited to hormonal therapy, mannitol, or neurotrophic factors 6,7. Even complex surgical decompression does not effectively improve neurological dysfunction and results in poor prognosis 8,9. Thus, comprehensively understanding SCI pathogenesis and exploring effective drug treatment targets have become a research focus.

Secondary injury processes are the most critical pathological changes following SCI. They include tissue loss, neuroinflammation, reactive oxygen species (ROS) accumulation, and neuronal cell death 10,11. Understanding and mediating the forms of neuronal cell death are important for alleviating neural tissue damage and promoting its regeneration 12. However, previous studies have focused on neuronal or oligodendrocyte death processes, with little attention given to the death of astrocytes, which are the most abundant and neuroprotective cells in the nervous system 13,14. Investigating the mechanisms that might be used to inhibit astrocyte death following SCI could provide new strategies for alleviating secondary injury and promoting functional recovery.

Necroptosis is a type of programmed cell death that combines apoptotic and necrotic features 15-17. Pivotal events include activation of receptor-interacting protein kinase (RIPK) 1 and RIPK3, which form a necrosome complex 18. This leads to mixed lineage kinase domain-like protein (MLKL) phosphorylation, which disrupts the plasma membrane and causes cell death 19,20. Necroptosis is initiated by death receptors, such as tumor necrosis factor receptor, and is independent of caspase activity 21,22. It plays significant roles in various pathological conditions, including neurodegenerative diseases, infections, inflammation, and ischemic injuries 23,24. However, whether astrocytes undergo necroptosis following SCI and the regulatory mechanisms involved in this process remain unclear.

Mitochondria are present in most neuronal cells, including astrocytes, where they facilitate aerobic respiration and energy production 25. ROS accumulation can compromise mitochondrial function and cause loss of mitochondrial membrane potential (MMP), leading to a cascade of events that trigger mitophagy 26,27. This loss prevents the import of PTEN-induced kinase 1 (PINK1) into the inner mitochondrial membrane, causing its accumulation on the outer membrane and subsequent recruitment of Parkin 28. Parkin ubiquitinates outer mitochondrial membrane proteins, marking the damaged mitochondria for degradation 29. Autophagy receptors, such as p62, nuclear dot protein 52, and optineurin, recognize these ubiquitinated proteins and link them to microtubule‑associated protein light chain 3 (LC3) on autophagosomal membranes 30-32. Damaged mitochondria are engulfed by autophagosomes, which mature and fuse with lysosomes to form autolysosomes. Mitochondrial components are degraded by lysosomal hydrolases within autolysosomes, leading to their recycling 33. However, excessive ROS generated during secondary injury prevent effective clearance of the damaged mitochondria via the mitophagy pathway. Uncleared damaged mitochondria simultaneously produce even more ROS, creating a cycle that severely impairs physiological functions of astrocytes 34,35. Therefore, promoting mitophagy in astrocytes helps to maintain normal cellular functions. However, whether it aids in inhibiting astrocytic necroptosis remains unknown.

Fanconi anemia complementation group C (FANCC), a critical target gene in Fanconi anemia, has been extensively investigated in hematologic disorders and shown to mitigate SCI-induced neuroinflammation 36. There are presently no reports on whether FANCC can regulate mitophagy and necroptosis in astrocytes following SCI. In the present study, astrocyte-specific FANCC conditional knockout (*Fancc^fl/fl^-GFAP-Cre*) mice were generated to investigate the action of FANCC on activating astrocytic mitophagy and inhibiting necroptosis following SCI, as well as the potential molecular mechanisms involved in these processes. In particular, 5D proteomics in conjunction with protein interaction assays demonstrated that FANCC can directly interact with TANK-binding kinase 1 (TBK1) to promote mitophagy. FANCC agonist poliumoside was intraperitoneally injected into SCI mice to explore the impact of FANCC activation on promoting neural functional recovery *in vivo*. This study was aimed at elucidating the molecular mechanisms underlying FANCC-mediated mitophagy activation and necroptosis inhibition in astrocytes, to provide novel insights into potential therapeutic strategies for SCI.

## Methods

### Animals

*Fancc^fl/fl^* and *GFAP-Cre* mice were purchased from Cyagen Biosciences (Suzhou, China), and their offspring were genotyped to establish astrocyte-specific FANCC conditional knockout (cKO) mice (*Fancc^fl/fl^-GFAP-Cre*). Briefly, exons 5 and 6 were selected as the cKO region, which contained a 179 bp coding sequence. To engineer the targeting vector, we generated homologous arms and the cKO region with polymerase chain reaction (PCR) using Bacterial Artificial Chromosome clone RP23189B9 as the template. The ribonucleoprotein and targeting vector were co-injected into fertilized eggs for cKO mouse production. The targeting strategy and gRNA sequences are shown in Figure S1 and Table S1. Wild-type (WT) C57BL/6J mice (8 weeks old, 20-30 g) were provided by Huachuang Sino Pharma Tech Co. Ltd. (license no. SCXK 2020-0009, Taizhou, China). All mice were housed in an environment at a constant temperature and humidity with a 12-h light/dark cycle. They were provided with a standardized, commercially available, and specifically formulated rodent chow and uncontaminated water. All of the mice were adapted to the experimental environment before the study procedures were carried out.

### Establishment of SCI mouse models

Eight-week-old *Fancc^fl/fl^-GFAP-Cre* (n = 6) and matching *Fancc^fl/fl^* (n = 6) mice underwent spinal cord contusion and laminectomy procedures as previously described 37. After safely anesthetizing the mice with a ketamine/xylazine mixture, an incision was made and paraspinal muscles were removed to expose the T10 spinous process. A laminectomy was then performed to expose the spinal cord. A spinal cord impactor (68097, RWD, Shenzhen, China) was utilized to induce a contusion injury in the exposed area of the spinal cord. Successful model construction was indicated by the formation of a hematoma at the impact site, tail flick reflex, and hind limb paralysis post-injury. A simple laminectomy procedure was performed on the sham group mice. Due to the possibility of bladder dysfunction caused by SCI, the mice were assisted with urination over the course of 1 week by gently squeezing their bladders to promote urination recovery and prevent retention. In addition, the SCI mice were raised to keep the wound clean and dry and their dressings were regularly changed to prevent infection.

### Intraperitoneal administration of poliumoside

To determine the potential therapeutic benefits of FANCC in SCI, a virtual screening FANCC agonist (poliumoside, MedChemExpress, Weehawken, NJ, USA) was injected intraperitoneally into sham or SCI mice at a concentration of 20 or 40 mg/kg once daily for 1 week. Mice receiving an equal volume of saline were used as the controls. The mice were euthanized 28 days post-injury (dpi) and their spinal cords were collected for further analysis.

### Histological analyses

Hematoxylin and eosin (HE), Masson's, Nissl, and Luxol Fast Blue (LFB) staining methods were used to evaluate the neuropathological and morphological changes of injured spinal cords in mice according to the manufacturer instructions for each detection kit (Servicebio, Wuhan, China). For HE staining, the sections were stained with hematoxylin to color the nuclei blue and then with eosin to color cytoplasmic and extracellular components pink. In Masson's staining, Weigert's hematoxylin stained nuclei blue-black, Biebrich scarlet-acid fuchsin stained cytoplasm and muscle fibers red, and aniline blue stained collagen fibers blue. The sections were then differentiated with phosphomolybdic-phosphotungstic acid, dehydrated, cleared with xylene, and mounted with a coverslip. For Nissl and LFB staining, after staining with toluidine blue to color Nissl bodies in neurons blue or staining with LFB solution to color myelin sheaths blue, the sections were rinsed, dehydrated, cleared with xylene, and mounted with a coverslip, which allowed detailed examination of neurons and myelin distribution. All of the stained sections were observed with a florescence microscope (Leica, Oskar, Germany).

### Behavioral/locomotor assessment

Motor function recovery in SCI mice was evaluated based on the Basso Mouse Scale (BMS), gait analysis system, and hindlimb reflex scoring. For BMS analysis, the mice were scored according to their motor function on a scale from 0 to 9, where 0 indicated complete lack of motor function with total paralysis of the hind limbs, and 9 indicated completely normal gait, indistinguishable from healthy mice. The mice were placed in an open field (gait analysis box) to allow free movement and assess their hind limb motor ability, gait coordination, and stability. The gait data were collected through the DigiGait imaging and analysis system (Mouse Specifics, Framingham, MA, USA) to evaluate the gait changes 38. The mice were placed on the treadmill of the gait analysis system and allowed to adapt to the environment to avoid stress and unnatural gait. The camera system and pressure sensors were then activated to record mouse movements. Computer software was used to process the captured images and pressure data to extract gait parameters and perform analysis and scoring. For the hindlimb reflex score, mice were suspended by the tail at a height of ~30 cm for 14 s. Their posture was rated based on the following criteria: 0, normal; 1, failure to extend hindlimbs; 2, hindlimb clasping; and 3, hindlimb paralysis 39. Two independent graders blinded to the experimental conditions evaluated the SCI mice three times and recorded the average scores.

### Bulk RNA sequencing (RNA-Seq) bioinformatics analysis

The RNA-Seq dataset GSE5296 was downloaded from the Gene Expression Omnibus (GEO) database and included samples from 36 sham-operated mice and 36 mice in the acute SCI phase within 7 dpi. The sequenced tissues were obtained from the T8 spinal cord region. Data normalization and differential gene expression analysis were conducted using the limma package in R. Genes with a p value of <0.05 and an absolute log_2_ fold change (FC) of ≥1 were classified as differentially expressed 40. Lasso regression was used to identify genes specifically associated with SCI among the differentially expressed genes. Lasso regression results were validated using logistic regression, ensuring the robustness and reliability of the identified SCI-related genes. The significance and importance of SCI-related genes were further assessed using a random forest model. Consensus clustering was utilized to identify molecular SCI subtypes based on the Lasso regression results. Gene Ontology enrichment analysis was conducted using the ClusterProfiler package in R to explore the biological processes linked to each SCI molecular subtype 41.

### Single-nucleus (sn) RNA-Seq bioinformatics analysis

The snRNA-Seq dataset utilized in the present study was based on the study by Skinnider *et al.* 42 and consisted of 151712 cells from the T9 spinal cord of mice in the acute SCI phase within 1 month (m). Data normalization and preprocessing were performed using the Seurat package's standard pipeline in R 43. The Seurat package was used to process the raw data, including quality control, normalization, scaling, and identification of highly variable genes. The weighted nuclear density algorithm was applied to calculate ROS scores according to the consensus clustering results based on the bulk RNA-Seq dataset, particularly focusing on the SCI subtype Cluster2. Astrocytes were subsequently extracted and subjected to further subclustering at a resolution of 0.3, resulting in the identification of five distinct astrocyte subpopulations. The Monocle3 package was used to track the differentiation trajectory of astrocyte populations. To achieve a more refined temporal resolution of astrocyte dynamics, we subdivided the astrocyte into 9 distinct subpopulations at a resolution of 0.5. In parallel, marker genes for 16 distinct cell death modalities were collected from the GeneCards database 44 using a threshold of "Relevance score ≥ median*2 (Relevance score)". The investigated cell death modalities included necroptosis, apoptosis, ferroptosis, pyroptosis, autophagy, netosis, immunogenic cell death, cuproptosis, entotic cell death, parthanatos, paraptosis, methuosis, netotic cell death, oxeiptosis, entosis, and alkaliptosis. The weighted nuclear density algorithm was used to calculate cell death scores for each of these 16 modalities across the snRNA-Seq data. Finally, cell-cell communication analysis was performed with the Cell-Cell Contact database using the CellChat package 45.

### Cell culture

Primary astrocytes and neurons were extracted from neonatal or fetal mice. For astrocyte extraction, 1-3-day specific-pathogen-free C57BL/6J mice were euthanized by immersion in 75% ethanol for 5 min. The brain tissue was dissected on ice under a stereo microscope (77001S, RWD) and the microvessels and meninges were removed from the cortex. The dissected brain tissue was cut into pieces using scissors, resuspended in 0.25% trypsin, and transferred to a culture dish. The tissue cell suspension was filtered through a cell strainer, collected, and centrifuged at 1,300 rpm for 5 min. The culture medium was changed after 24 h and every 3 days thereafter, and the bottom astrocyte layer was shaken at 200 rpm and 37℃ for 12 h and digested with trypsin. The astrocytes were then shaken again at 200 rpm and 37℃ for a further 12 h three days later and placed back in the cell culture incubator for cultivation. The astrocytes were maintained in Dulbecco's modified Eagle's medium (KeyGEN, Nanjing, China) supplemented with 10% fetal bovine serum (Gibco, Grand Island, NY, USA) until further experiments.

For neuron extraction, the cortex was dissected from 16-18-day fetal mice and minced into small pieces. The tissue was enzymatically digested with papain for 15-30 min at 37℃ and gently triturated to create a single-cell suspension. The suspension was filtered through a 70-m cell strainer to remove debris and centrifuged at 1,000 rpm to pellet the cells. The cells were then resuspended in Dulbecco's modified Eagle's medium with 10% fetal bovine serum and plated onto six-well plates coated with poly-D-lysine. Cell adhesion was observed after 4 h and the medium was replaced with Neural Basal Medium (Gibco) supplemented with 1 GlutaMAX (Gibco) and 2% B27 (Gibco). The medium was changed every 2 days thereafter. The neurons were allowed to adhere and incubated at 37℃ with 5% CO_2_, with the medium changed regularly.

### Cell transfection and treatment

Astrocytes were transfected with lentivirus shRNA-FANCC (KD-FANCC) or FANCC (OE-FANCC) at 10^7^ TU/mL supplemented with HitransG A (Shanghai Genechem Co. Ltd.) for 12 h. A total of 2.5 μg of plasmids with either full-length TBK1 (Flag-TBK1) or deleted TBK1 (Flag-Mut) (GeneChem) was co-transfected with a plasmid containing HA-FANCC (GeneChem) into HEK293T cells (Procell Life Technology, No. CL-0005, Wuhan, China). After 48 h of incubation at 37°C, the cells were collected for co-immunoprecipitation (Co-IP) analysis. To induce astrocyte necroptosis, tumor necrosis factor-α (TNF-α, 100 ng/mL), lipopolysaccharides (LPSs, 4 μg/mL), and z-VAD (20 μM) (TLZ) were used as described previously 46 and added to the culture medium for 48 h.

### Co-culture of astrocytes and neurons

A co-culture strategy was used to study the effect of necroptotic astrocytes on neuronal survival. Astrocytes and neurons were isolated and cultured in accordance with the aforementioned description. Necroptosis was induced in astrocytes using TLZ. After 24 h, culture medium from necroptotic astrocytes transfected with OE-FANCC or KD-FANCC was transferred into the neuron culture dish, and the effects of necroptotic astrocytes on neuronal survival were observed.

### Quantitative real-time PCR (qPCR)

Total RNA was extracted from cells using TRIzol reagent (YIFEIXUE BioTech, Nanjing, China). For RNA samples, a reverse transcription step was required to generate cDNA. The qPCR reaction system, including template nucleic acid, specific primers, SYBR Green fluorescent dye, dNTPs, buffer, and Taq DNA polymerase, was prepared. The sample nucleic acid was mixed thoroughly with the prepared reaction system in qPCR plates. The reaction system was placed in the qPCR machine for thermal cycling (Roche LightCycler 480, Roche, Basel, Switzerland). Data were analyzed using a standard curve. The ΔΔCt method was used to determine relative mRNA expression levels. The primer information is provided in Table S2.

### Western blotting

The protein samples were prepared by lysing the cells or tissues in a lysis buffer containing protease inhibitors. The protein concentration was quantified with bicinchoninic acid assays (KeyGEN). The proteins were separated by SDS-PAGE and transferred onto a polyvinylidene difluoride membrane, which was then incubated with a primary antibody specific to the target protein, followed by washing to remove the unbound antibody. Next, the membrane was incubated with a secondary antibody and washed again. The protein bands were detected using enhanced chemiluminescence and visualized with an imaging system (Syngene, Cambridge, UK). The band intensity was analyzed using ImageJ software (National Institutes of Health, Bethesda, MD, USA) to quantify the protein expression levels. Complete antibody details are provided in Table S3.

### Immunofluorescence (IF) analyses and fluorescence localization

The cells were fixed with paraformaldehyde and permeabilized with Triton X-100. Non-specific binding was blocked with a blocking buffer (Beyotime, Nanjing, China). After incubating with a primary antibody specific to the target protein and then with a fluorescently labeled secondary antibody, unbound antibodies were removed and the cells were incubated with 4,6-diamino-2-phenyl indole for nuclear staining. To detect autophagosomes and autolysosomes, cells were precultured in six-well plates and transiently transfected with GFP-mRFP-LC3 adenovirus at a multiplicity of infection of 20 (autophagy probe; HB-AP210 0001, Hanbio, Shanghai, China). Fluorescence images were captured 24 h post-infection using a confocal laser scanning microscope (Nikon Eclipse Ti, Tokyo, Japan). For mitochondrial localization, cells were incubated with 100 nM Mito-Tracker Red (Hanbio) for 30 min at 37C and the fluorescent organelles were examined using a confocal microscope. For IF tissue staining, spinal cord samples were fixed with paraformaldehyde and embedded in paraffin. Subsequent steps were similar to those described for the cellular IF protocols. High-resolution images were obtained and co-staining and localization of different indicators was visualized.

### Neuronal morphology analysis

Sholl analysis of neurons involves quantifying neuronal dendritic arbor complexity 47. To accomplish this, neurons were first stained or labeled to clearly visualize their dendrites. Digital neuron images were captured using a microscope (Leica). Concentric circles were drawn around the soma (cell body) at regular intervals using image analysis software. The number of dendritic intersections with each circle was counted starting from the innermost circle and moving outward and then plotted to generate a Sholl profile showing the number of intersections as a function of the distance from the soma.

### Transmission electron microscopy (TEM)

The cells were centrifuged and then treated with electron microscopy fixative (G1102, Servicebio) and 0.1 M phosphate buffer (pH 7.4) at 4℃. Then, 1% agarose solution was prepared by heating it until it dissolved. Next, the cell pellet was transferred into an Eppendorf tube and suspended in the agarose solution before it solidified. The sample was fixed with 1% osmium tetroxide in 0.1 M phosphate buffer at room temperature for 2 h in the dark and dehydrated using ethanol followed by 100% acetone twice for 15 min each time. After infiltration and embedding, the sample was placed in a 60℃ oven for 48 h to polymerize. Ultrathin sections were cut using an ultramicrotome and collected on 150-mesh formvar-coated copper grids. The grids were stained with 2.6% lead citrate in the dark for 8 min, washed three times with ultrapure water, and dried with filter paper. The sections were observed using a transmission electron microscope (Hitachi, Tokyo, Japan) and their images were recorded.

### Flow cytometry (FCM)

The cells were harvested and washed with cold phosphate-buffered saline (PBS) and stained with 5 µL of annexin fluorescein isothhiocyanate (V-FITC) and propidium iodide (PI, Beyotime), followed by incubation in the dark for 5 min at room temperature. After incubation, the cells were washed with the binding buffer to remove excess staining solution, resuspended in binding buffer, and analyzed immediately using a flow cytometer (FACSVerse 8, BD Biosciences, NJ, USA). During analysis, the cells undergoing necroptosis exhibited diffuse PI staining and intense fluorescence signals, while annexin V-positive/PI-negative cells were considered apoptotic.

### ROS and MMP measurement

The cells were treated with ROS-sensitive fluorescent dyes Cellrox-green and Mitosox-red (5 μmol/L, C10444, M36007, Thermo Fisher Scientific, Waltham, MA, USA) and incubated for 30 min at 37C in the dark. After incubation, the cells were washed to remove excess dye. The cells were then analyzed immediately using fluorescence microscopy. For MMP detection, the JC-1 staining solution was prepared by diluting JC-1 dye in culture medium (ab113850, Abcam, Cambridge, MA, USA). The cells were incubated with the JC-1 staining solution for 20 min at 37C in the dark. After staining, the cells were washed with PBS to remove excess dye. JC-1 formed aggregates emitting red fluorescence in healthy cells with intact MMP and remained in monomeric form emitting green fluorescence in cells with compromised MMP.

### Co-IP assay

After treatment, astrocytes were washed three times with cold PBS and incubated with protein lysis buffer at 4C for 30 min. The lysates were then centrifuged (12,000 *g*, 10 min, 4°C) to obtain the supernatant. Next, 80 µL of the supernatant was reserved as the input sample, while the remaining supernatant was incubated with specific target protein and IgG antibodies (as a negative control) along with Protein G Sepharose beads (GE Healthcare, Stockholm, Sweden) overnight at 4°C. After capturing the protein complexes, the beads were washed extensively to remove nonspecifically-bound proteins and contaminants. The IP protein complex was eluted from the beads using an elution buffer under denaturation. Finally, the eluted proteins were analyzed using western blotting to detect and characterize the interacting proteins.

### Label-free proteomics and bioinformatics analyses

After the Co-IP analysis, 20 µg of protein was mixed with the loading buffer and subjected to 12% SDS-PAGE followed by Coomassie Brilliant Blue staining. In addition, 100 µg of protein from each sample was mixed with dithiothreitol to a final concentration of 100 mM, heated in a boiling water bath for 5 min, and cooled to room temperature. The peptides were analyzed using a nanoscale liquid chromatography tandem mass spectrometry (MS/MS) system (Easy-nLC 1200, Thermo Fisher Scientific) coupled with a Q-Exactive mass spectrometer (Thermo Fisher Scientific). MS/MS analysis was performed in data-dependent acquisition mode, scanning precursors in the range of 350 to 1600 m/z.

For the label-free proteomics data analysis, the data were filtered to include only those samples where at least half of the replicates did not have any missing values. These selected data were then log_2_-transformed. Differential protein expression was analyzed using the limma package in R. Lasso regression was applied to the differentially expressed proteins to identify those linked to SCI. Based on the identified SCI-related proteins, Gene Ontology 48 and Kyoto Encyclopedia of Genes and Genomes (KEGG) 49 enrichment analyses were performed using the ClusterProfiler package in R.

### Molecular docking and virtual drug screening

The protein-protein docking program ZDOCK was used to effectively explore the rigid-body search space of protein-protein docking positions employing fast Fourier transform. It continuously performed local searches and repeated iterations to find the optimal molecular docking conformations for FANCC and TBK1. Schrödinger Maestro 11.4 software was used for virtual drug screening and PyMol software was used for three-dimensional (3D) plotting. The predicted 3D structure of mouse FANCC (AlphaFold ID: AF-P50652-F1) was downloaded from the AlphaFold website. The 2D compound structures from the Life Chemicals 50K Diversity Library (containing ~50,200 compounds) and the MCE Bioactive Compound Library (containing ~17,000 compounds) were processed using the Schrödinger LigPrep Module for hydrogen addition, energy minimization, and conversion to 3D structures for virtual screening. The Virtual Screening Workflow module was used for virtual screening, where the prepared compounds were imported and molecular docking was performed using the Glide module. The compound binding affinity and structure were manually reviewed, and the small molecules with strong binding affinity to mouse FANCC protein were selected.

### Statistical analysis

Data were presented as mean ± standard deviation (SD). Group comparisons were conducted using the two-tailed Student's *t*-test for pairwise comparisons and two-way analysis of variance followed by Tukey's *post hoc* test for multiple groups. Analyses were performed using R software (version 4.2.0) and GraphPad Prism 9.3 (La Jolla, CA, USA). Statistical significance was defined as *p < 0.05, **p < 0.01, ***p < 0.001, ****p < 0.0001, and no significance (n.s.).

## Results

### Astrocytic FANCC expression is a protective factor after SCI

**Figure 1:**
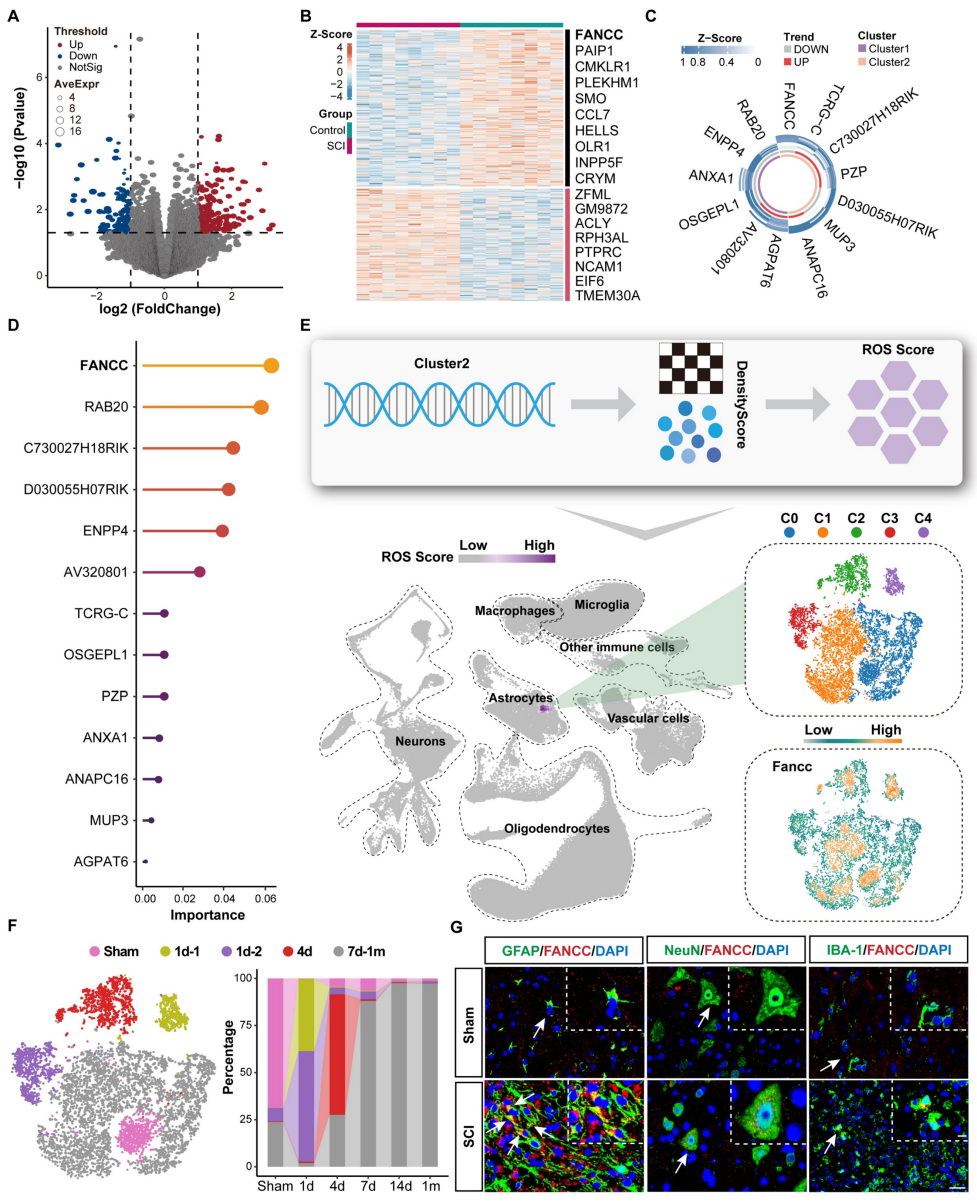
** FANCC expression is closely associated with astrocytes after SCI.** (**A**) Volcano plot of gene expression after SCI. (**B**) Heatmap indicating differentially expressed genes. (**C**) Molecular subtype analysis of 13 genes using consensus clustering in unsupervised machine learning, and named as Clusters 1 and 2. (**D**) The random forest model, through supervised machine learning, was used to determine gene weights. (**E**) ROS Score based on the weighted nuclear density algorithm and the expression of FANCC in five subsets of astrocytes. (**F**) Proportions of five astrocyte states at different time points. (**G**) Representative IF labeling images of GFAP, NeuN, IBA-1 (green), and FANCC (red) in the spinal cord for Sham group and 7 dpi group; scale bar = 20/10 μm.

A public dataset from the GEO database containing bulk-RNA-Seq data for injured and normal mouse spinal cords was used to carry out differential gene expression analysis after quality control was performed (Table S4). The volcano plot with cutoff values of p<0.05 and absolute log_2_^FC^≥1 showed 187 upregulated genes in red and 1,246 downregulated genes in blue after SCI (Figure 1A). The differentially expressed genes were then extracted and their expression levels plotted as a heatmap (Figure 1B). Lasso regression for penalized dimension reduction was used to identify genes closely associated with SCI (Figure S2A). A logistic regression risk model was constructed based on the Lasso regression results and identified 13 genes related to SCI. Among them, seven were protective (odds ratio <1), and six were risk genes (odds ratio >1) (Figure S2B and Table S5). Molecular subtype analysis was subsequently performed using consensus clustering to clarify the molecular characteristics of 13 SCI-related genes as an unsupervised machine learning method. The results indicated that these 13 genes could be divided into two subtypes at the molecular level, which were named Clusters 1 and 2 (Figure 1C and Table S6). Enrichment analysis revealed that Cluster 1 was enriched in biological functions related to wound healing, while Cluster 2 was enriched in biological functions related to oxidative stress. Therefore, the seven genes in Cluster 2 potentially represented the ROS subtype (Figure S2C and Table S7). A random forest model, a supervised machine learning method, was used to calculate the weights of the 13 genes, and FANCC had the highest weight (Figure 1D). To further identify genes associated with SCI with high robustness, we further identified six genes by using a threshold of weighted score ≥ median*2 (weighted score). Co-expression analysis between Clusters 1 and 2 with the random forest model was performed to determine ROS-associated genes after SCI (Figure S2D). FANCC, a protein-coding RNA, was found to potentially play a major role in the ROS phenotype after SCI (Figure S2D). To further clarify the cell-specific functions of FANCC after SCI, we used the weighted nuclear density algorithm based on the genes in Cluster 2 to obtain the ROS Score after quality control, dimension reduction, and other preliminary processing steps were performed on the snRNA-Seq dataset (Figure 1E). The ROS Score was highest in the astrocytes, underscoring the need to focus on their biological function after SCI (Figure 1E and Table S8). We next divided the astrocytes into five subsets, C0-C4, according to biological state. FANCC was widely expressed in astrocytes in all subsets, thus indicating that the changes in FANCC expression in astrocytes influenced the secondary pathological process after SCI (Figure 1E).

Through differentiation trajectory analysis of astrocytes, we further identified three distinct activation phases corresponding to 1d, 4d, and 7d to 1m post-injury (Figure S3A). To resolve temporal dynamics with enhanced resolution, we stratified astrocytes into nine transcriptionally distinct subclusters (S0-S8) (Figure S3B). Initial temporal categorization revealed four injury progression states: Sham, 1d, 4d, and 7d-1m. Notably, spatial segregation of two subclusters in t-SNE plot within the 1d group prompted their subdivision into 1d-1 and 1d-2 subcategories (Figure 1F). Cross-referencing these five temporally defined states with original clusters established the following mapping: Sham (S6), 1d-1 (S8), 1d-2 (S4), 4d (S3), and 7d-1m (S0/S1/S2/S5/S7) (Figure 1F). Dynamic remodeling of these states across the injury timeline implied temporally specialized functional roles. Functional enrichment profiling revealed state-specific biological signatures: Sham astrocytes maintained homeostatic functions including synaptic modulation, while 1d-1 subclusters showed ribosomal biogenesis activation. The 1d-2 population demonstrated pattern recognition receptor signaling upregulation. Transmembrane ion transport machinery dominated the 4d phase, whereas 7d-1m clusters exhibited coordinated neuroinflammatory signaling and redox system activation (Figure S3C). Additionally, ROS Score confirmed significant oxidative stress escalation in 7d-1m-phase astrocytes compared to other states (Figure S3D). Collectively, these findings suggested that astrocytes entered a highly activated inflammatory and oxidative stress state at 7 dpi. In addition, when co-localized with relevant neural cells such as neurons, microglia, and astrocytes in the sham group and 7 dpi group, FANCC was predominantly expressed in astrocytes (Figure 1G).

Remarkably, IF results of spinal cord tissue within 1m after injury revealed that FANCC expression in astrocytes peaked at 7 dpi (Figure S3E-F). These findings were consistent with the previous extensive bioinformatics analysis, indicating that FANCC played a significant role in regulating the biological function and state of astrocytes following SCI.

### FANCC deficiency in astrocytes exacerbates neuropathological degeneration and motor dysfunction in SCI mice

**Figure 2:**
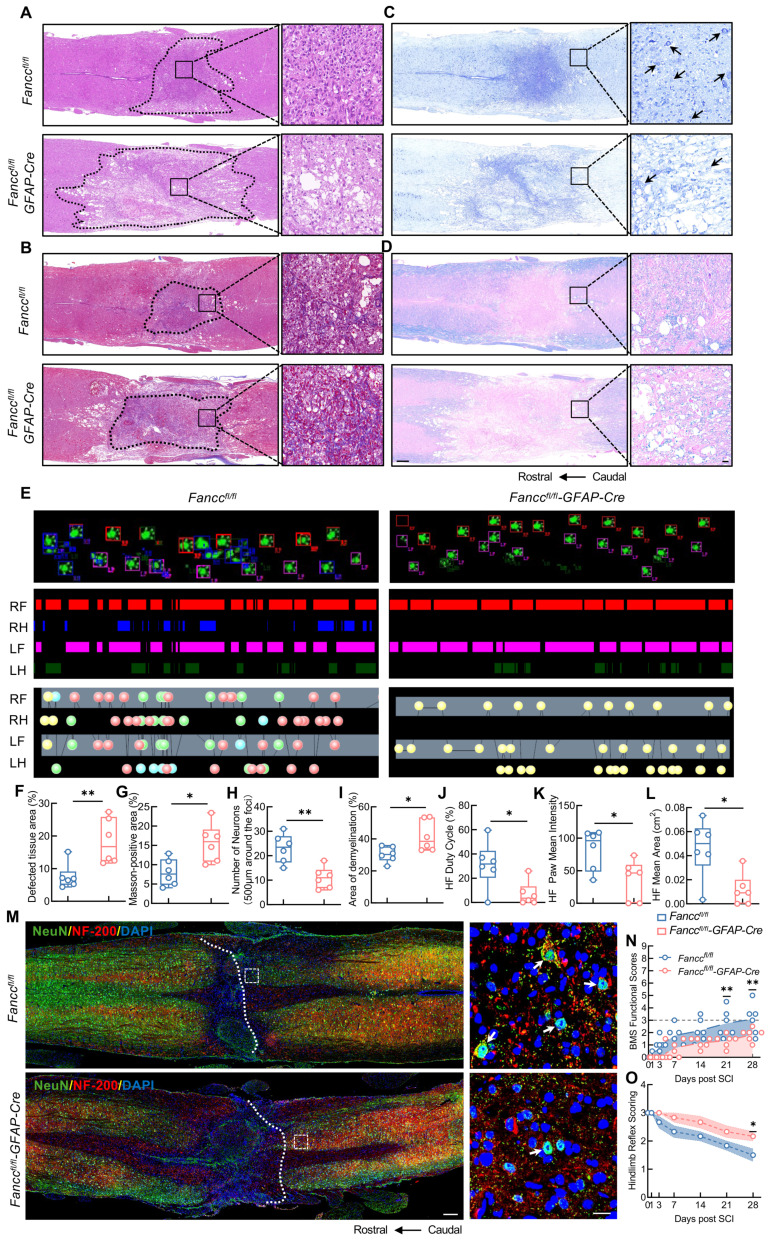
** Deficiency of FANCC exacerbates neuropathological degeneration and motor dysfunction in SCI mice.** (**A**) HE staining of injured spinal cord obtained at 28 dpi in *Fancc^fl/fl^* and *Fancc^fl/fl^-GFAP-Cre* mice (n = 6); scale bar = 250/20 μm. (**B**) Masson's staining of the injured spinal cord obtained at 28 dpi in *Fancc^fl/fl^* and *Fancc^fl/fl^-GFAP-Cre* mice (n = 6); scale bar = 250/20 μm. (**C**) Nissl staining images of the injured spinal cord at 28 dpi in *Fancc^fl/fl^* and *Fancc^fl/fl^-GFAP-Cre* mice (n = 6); scale bar = 250/20 μm. (**D**) LFB staining of the injured spinal cord at 28 dpi in *Fancc^fl/fl^* and *Fancc^fl/fl^-GFAP-Cre* mice (n = 6); Scale bar = 250/20 μm. (**E**) Representative footprint images were recorded in *Fancc^fl/fl^* and *Fancc^fl/fl^-GFAP-Cre* mice after SCI (n = 6). The panels are footprints of the hindpaw reflected by green LED light and the colored bands represent standing time of each foot. (**F-I**) Quantitative analysis of the affected area, Masson-positive area, number of neurons and the demyelinated area at 28 dpi. (**J-L**) The duty cycle, paw mean intensity, and mean area of hindlimb footprints were analyzed by DigiGait software. (**M**) Representative immunofluorescence labeling images of NeuN (green) and NF200 (red) in the spinal cord in *Fancc^fl/fl^* and *Fancc^fl/fl^-GFAP-Cre* mice at 28 dpi; scale bar = 200/20 μm. (**N**) BMS score within 28 dpi in *Fancc^fl/fl^* and *Fancc^fl/fl^-GFAP-Cre* mice (n = 6). (**O**) Hindlimb reflex score within 28 dpi in *Fancc^fl/fl^* and *Fancc^fl/fl^-GFAP-Cre* mice (n = 6). Data are presented as mean ± SD. Statistical significance was defined as *p < 0.05, **p < 0.01, ***p < 0.001, ****p < 0.0001, and no significance (n.s.).

The *Fancc^fl/fl^-GFAP-Cre* and *Fancc^fl/fl^* mice were used to investigate the role of FANCC in SCI development and prognosis. HE staining showed that *Fancc^fl/fl^-GFAP-Cre* mice, compared with compared *Fancc^fl/fl^* mice, had a larger local spinal cord lesion area that was characterized by loose and disorganized cell arrangement at 28 dpi (Figure 2A, F). Masson's staining showed that the collagen fibers in the injured spinal cords of *Fancc^fl/fl^-GFAP-Cre* mice were more intensely stained and more disorganized, indicating that FANCC deficiency exacerbated fibrotic scar formation after SCI (Figure 2B, G). In addition, Nissl and LFB staining both indicated that *Fancc^fl/fl^-GFAP-Cre* mice had fewer neurons around the injured spinal cord and a larger pathological demyelination area than *Fancc^fl/fl^* mice, thereby suggesting that astrocytic FANCC deficiency significantly affected neurological recovery in SCI mice (Figure 2C-D, H-I). The duty cycle, paw mean intensity, and footprint mean area of the hindlimbs observed in *Fancc^fl/fl^-GFAP-Cre* mice in DigiGait were significantly lower than those in the *Fancc^fl/fl^* group at 28 dpi (Figure 2E, J-L). In addition, the number of NeuN and NF200 co-expressing neurons in *Fancc^fl/fl^-GFAP-Cre* mice was significantly smaller at 28 dpi than observed in the *Fancc^fl/fl^* group (Figure 2M). BMS and hindlimb reflex scores were used to further evaluate motor function recovery in the two groups of mice after SCI. *Fancc^fl/fl^-GFAP-Cre* mice showed markedly lower BMS scores than *Fancc^fl/fl^* mice at 21 and 28 dpi (Figure 2N). Higher hindlimb reflex scores in the *Fancc^fl/fl^-GFAP-Cre* mice at 28 dpi clarified the negative influence of astrocytic FANCC deficiency on functional restoration after SCI (Figure 2O).

### FANCC inhibits astrocyte necroptosis after SCI

**Figure 3:**
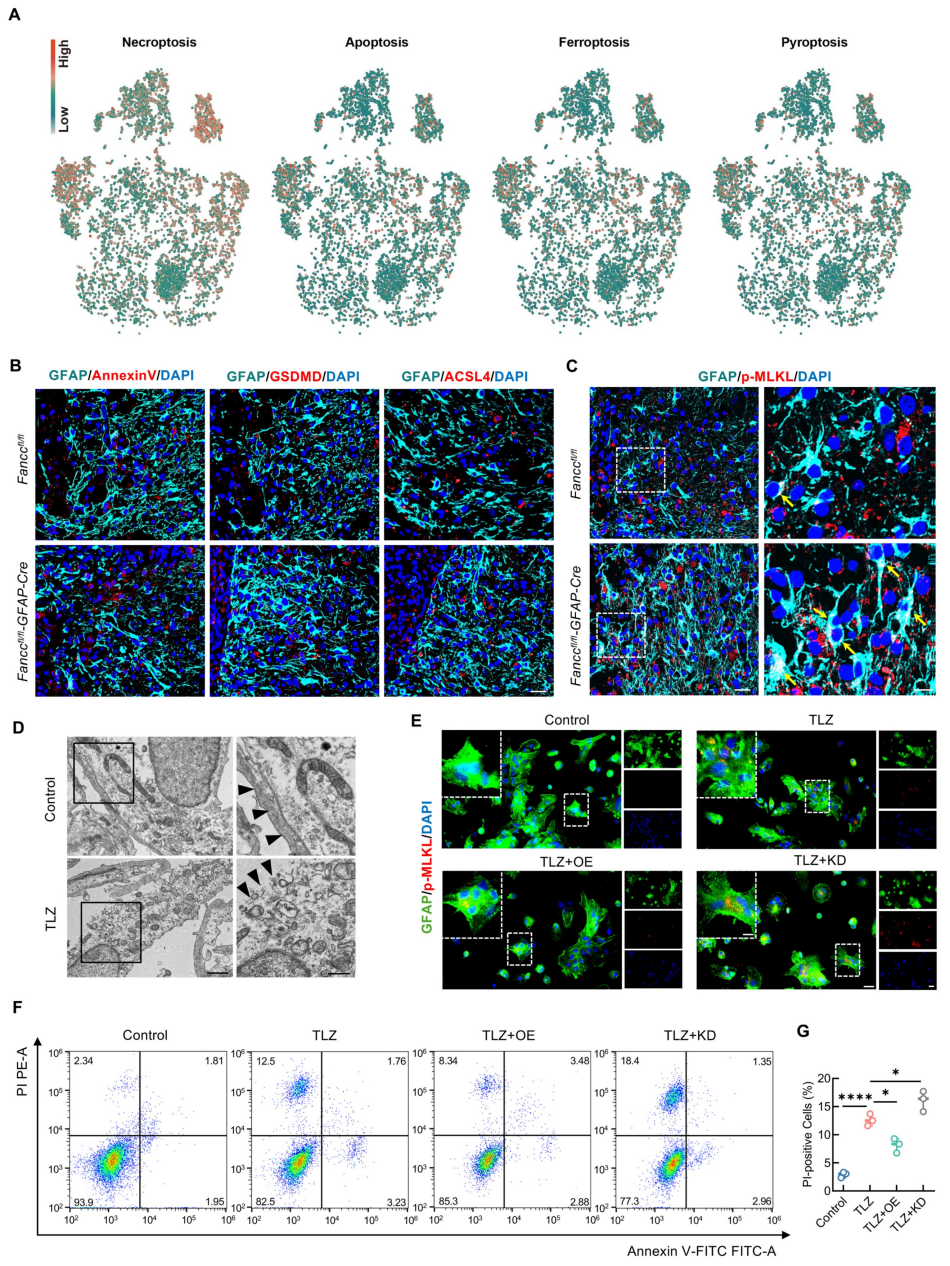
** FANCC inhibits astrocytic necroptosis after SCI.** (**A**) Scores of four cell death phenotypes (necroptosis, apoptosis, ferroptosis, and pyroptosis) calculated using the weighted nuclear density algorithm. (**B**) Representative IF images of annexinV, gasdermin D-N terminus, ACSL4 (red), and GFAP (green) in the spinal cord at 7 dpi; scale bar = 25 μm. (**C**) Representative IF images of p-MLKL (red) and GFAP (green) in the spinal cord at 7 dpi; scale bar = 25/10 μm. (**D**) TEM of TLZ-treated astrocytes; scale bar = 1 μm/500 nm. (**E**) Representative IF images of GFAP (green) and p-MLKL (red) in astrocytes treated with TLZ for 48 h after transfection with FANCC-OE or FANCC-KD; scale bar = 50/20/50 μm. (**F**) Representative images of FCM results labeled with PI and annexin-V-FITC in astrocytes treated with TLZ for 48 h after transfection with FANCC-OE or FANCC-KD. (**G**) Percentage of PI-positive astrocytes in different groups. Data are presented as mean ± SD. Statistical significance was defined as *p < 0.05, **p < 0.01, ***p < 0.001, ****p < 0.0001, and no significance (n.s.).

Several studies have shown that programmed cell death of neural cells is a critical process leading to poor outcomes following SCI 12. Our previous research found that FANCC inhibited microglial pyroptosis-mediated neuroinflammation. The GeneCards database was used to obtain genes related to 16 cell death phenotypes in astrocytes to further explore the role of FANCC in astrocytic death. The scores for the 16 cell death phenotypes were calculated with the weighted nuclear density algorithm. Necroptosis was highest in astrocytes after SCI (Figure 3A and Figure S4). Simultaneously, to further determine the type of astrocytic death mediated by FANCC, we performed co-staining for the markers of the four ROS-driven cell death phenotypes—necroptosis, apoptosis, ferroptosis, and pyroptosis—with GFAP in both *Fancc^fl/fl^* and *Fancc^fl/fl^-GFAP-Cre* SCI mice. The results showed no significant differences in astrocytes positive for annexin V, gasdermin D, and acyl-CoA synthetase long chain family member 4 (ACSL4) in the injured spinal cords of both groups (Figure 3B). However, the number and fluorescence intensity of astrocytes positive for the marker of necroptosis phospho (p)-MLKL significantly increased in *Fancc^fl/fl^-GFAP-Cre* mice after SCI (Figure 3C and Figure S5A). The expression of pivotal pathway necroptosis proteins RIPK1 and RIPK3 were further examined in *Fancc^fl/fl^-GFAP-Cre* SCI mice. FANCC deficiency resulted in significantly greater RIPK1 and RIPK3 expression in astrocytes than observed in *Fancc^fl/fl^* mice, thereby suggesting that FANCC inhibited necroptosis in astrocytes *in vivo* (Figure S5B-E). Primary astrocytes were extracted to investigate the inhibitory effect of FANCC on astrocyte necroptosis *in vitro* (Figure S5F). TLZ was used to induce necroptosis in astrocytes based on previous research 46. TEM revealed TLZ-stimulated astrocyte disintegration and plasma membrane rupture, cytoplasm granulation and densification, and organelle swelling, including mitochondria and the endoplasmic reticulum, which demonstrated that astrocytes underwent necroptosis (Figure 3D). IF showed that TLZ significantly increased p-MLKL expression, indicating necroptosis occurrence. In contrast, FANCC overexpression decreased the expression of p-MLKL, while FANCC knockdown further increased p-MLKL expression compared to the TLZ group levels (Figure 3E and Figure S5G). RIPK1 and RIPK3 under these treatment conditions also showed the same trend as p-MLKL (Figure S5H-K). FCM was used to investigate the effect of FANCC on necroptosis in astrocytes. FCM results showed that FANCC overexpression significantly decreased the proportion of PI-positive cells induced by TLZ, whereas this effect was reversed upon FANCC knockdown (Figure 3F-G). The above outcomes demonstrated that FANCC effectively inhibited astrocyte necroptosis following SCI both *in vivo* and *in vitro*.

### FANCC suppresses neuronal apoptosis mediated by necroptotic astrocytes

**Figure 4:**
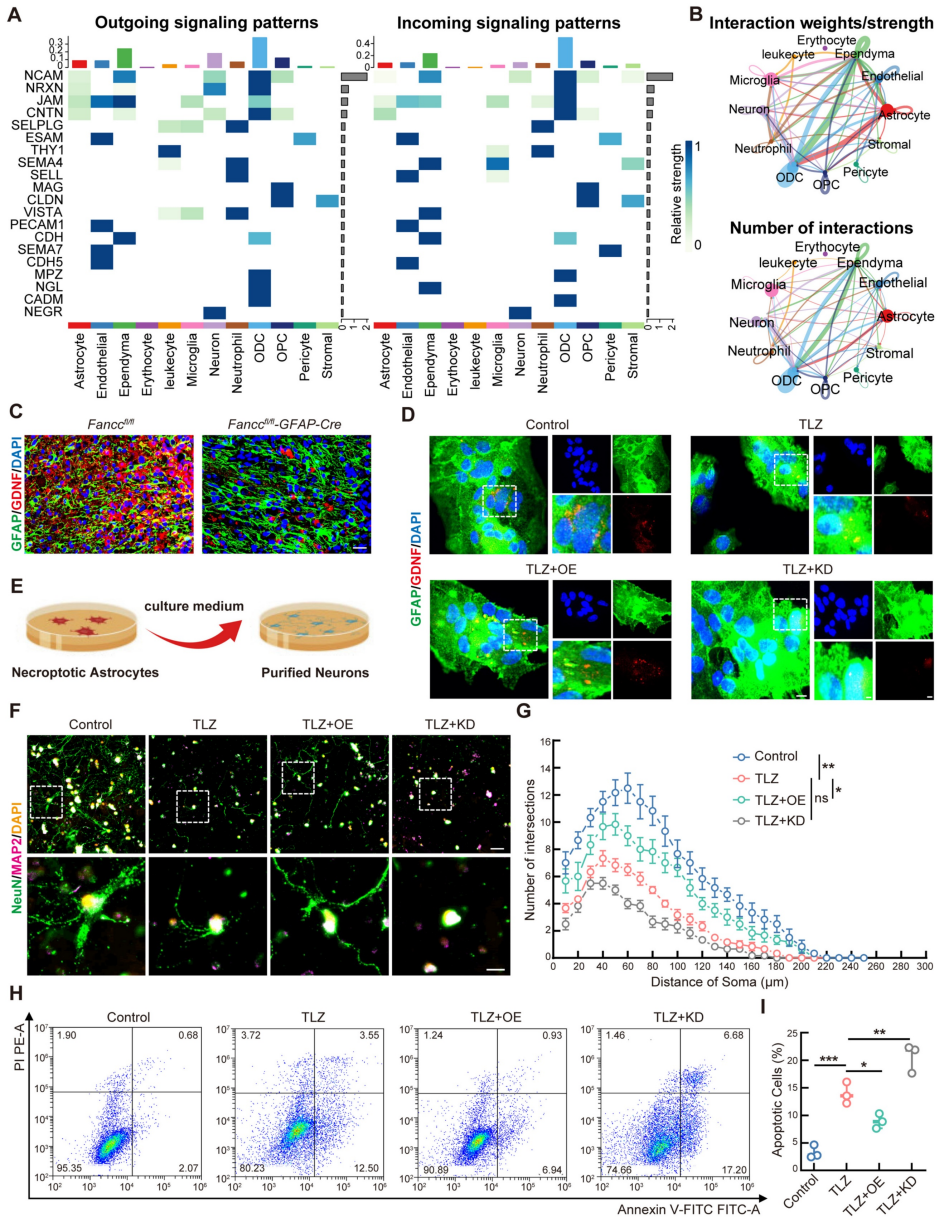
** FANCC suppresses neuronal apoptosis mediated by necroptotic astrocytes.** (**A-B**) Cell-cell communication analysis using the CellChat algorithm. (**C**) Representative IF images of GDNF (red) and GFAP (green) in the spinal cord at 7 dpi; scale bar = 50 μm. (**D**) Representative IF images of GFAP (green) and GDNF (red) in astrocytes treated with TLZ for 48 h after transfection with FANCC-OE or FANCC-KD; scale bar = 50/20/50 μm. (**E**) Schematic diagram of coculture model of astrocytes and neurons. (**F**) Representative IF images of NeuN (green) and MAP-2 (pink) in neurons induced by medium from necroptotic astrocytes for 48 h after transfection with FANCC-OE or FANCC-KD; scale bar = 40/10μm. (**G**) Quantification of intersections by Sholl analysis. (**H**) Representative images of FCM results, with labeling with PI and annexin-V-FITC in neurons induced by medium from necroptotic astrocytes for 48 h after transfection with FANCC-OE or FANCC-KD. (**I**) Percentage of apoptotic neurons in the indicated groups. Data are presented as mean ± SD. Statistical significance was defined as *p < 0.05, **p < 0.01, ***p < 0.001, ****p < 0.0001, and no significance (n.s.).

Astrocytes support neurons by secreting glial cell line-derived neurotrophic factor (GDNF), which promotes neuron survival, repair, and metabolic balance. However, necroptotic astrocytes lose the supportive role in neurons, leading to neuronal damage and apoptosis 50. The CellChat algorithm was utilized for cell communication analysis to further elucidate the cellular interactions following SCI, particularly those between astrocytes and neurons. The findings revealed the potential interactions between astrocytes and neurons (Figure 4A-B and Figure S6). Whether FANCC influenced the neurotrophic functions of astrocytes following necroptosis was investigated next. In contrast to observations in *Fancc^fl/fl^* SCI mice, astrocytic FANCC depletion significantly reduced GDNF expression by reactive astrocytes (Figure 4C). *In vitro*, FANCC overexpression increased GDNF expression in necroptotic astrocytes, whereas FANCC knockdown decreased it (Figure 4D). To study the impact of necroptotic astrocytes on neuronal survival, a co-culture model of astrocytes and neurons was constructed (Figure 4E). Sholl analysis was performed to assess whether astrocytic FANCC expression under necroptosis affected neuronal arborization. Analysis results revealed a greater number of intersections from the radial distance to the cell soma, indicating more complex dendritic arbors in the control group. In contrast, neurons treated with conditioned medium from TLZ-treated astrocytes exhibited a significant decrease in intersections. However, neurons exposed to FANCC-OE astrocytes showed a notable increase in intersections compared to those in the TLZ group (Figure 4F-G). FCM indicated that the proportion of apoptotic neurons treated with the conditioned medium from TLZ-treated astrocytes was significantly higher compared to that of the control group. However, the proportion of apoptotic neurons decreased when treated with the conditioned medium from FANCC-OE astrocytes. In contrast, FANCC knockdown caused a significant increase in neuronal apoptosis compared to that in the TLZ-only group (Figure 4H-I).

### FANCC maintains mitochondrial homeostasis in necroptotic astrocytes

**Figure 5:**
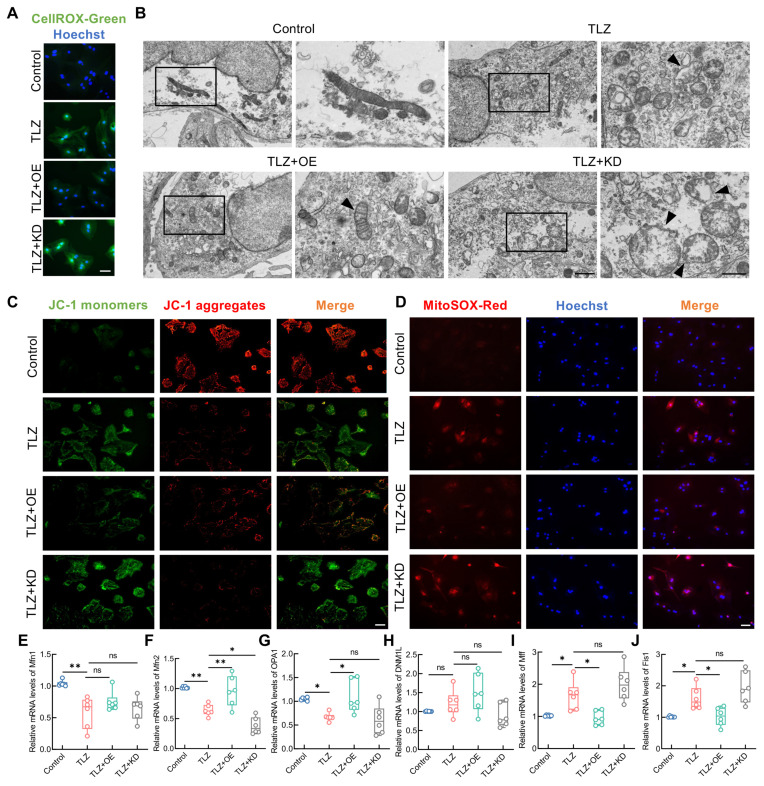
** FANCC maintains mitochondrial homeostasis in necroptotic astrocytes.** (**A**) Intracellular ROS levels were assessed using the CellRox Green probe; scale bar = 50μm. (**B**) Mitochondrial damage in necroptotic astrocytes observed under TEM in different groups; scale bar = 500 nm/250 nm. (**C**) MMP was determined using the JC-1 probe; scale bar = 100μm. (**D**) Mitochondrial ROS levels were assessed using the MitoSox Red probe; scale bar = 50μm. (**E-J**) Differentially expressed mitochondrial kinetic molecules assessed by qPCR. Data are presented as mean ± SD. Statistical significance was defined as *p < 0.05, **p < 0.01, ***p < 0.001, ****p < 0.0001, and no significance (n.s.).

As previously mentioned, necroptotic astrocytes exhibited ROS accumulation and mitochondrial damage. Therefore, whether FANCC could mitigate ROS accumulation and thereby maintain mitochondrial homeostasis was investigated. IF staining of intracellular ROS revealed that TLZ induced significant ROS accumulation in necroptotic astrocytes. This effect was reversed by FANCC overexpression, while FANCC deletion further exacerbated ROS release (Figure 5A). TEM analysis of astrocytes showed that TLZ induced mitochondrial swelling and the loss of typical elongated structure, leading to a more rounded or fragmented appearance. The mitochondrial membranes became permeabilized, which resulted in outer membrane rupture and release of the mitochondrial contents, accompanied by the inner membrane folds becoming disorganized. In contrast, necroptotic astrocytes treated with FANCC-OE showed partial recovery of mitochondrial morphology compared to those in the TLZ group (Figure 5B). The JC-1 kit was used to assess MMP in astrocytes. JC-1 in necroptotic astrocytes induced by TLZ failed to appear in its polymer form, leading to a decrease in MMP. However, an increase in MMP was observed after treatment with FANCC-OE. MMP decreased even further after FANCC knockdown, which indicated that FANCC could mediate mitochondrial homeostasis in necroptotic astrocytes (Figure 5C). Mitochondrial ROS levels were then examined in necroptotic astrocytes under various treatment conditions. Although TLZ significantly increased mitochondrial ROS levels, FANCC overexpression significantly reduced the high levels of mitochondrial ROS induced by TLZ. In addition, FANCC expression inhibition further increased mitochondrial ROS levels (Figure 5D). Mitochondrial fusion and fission are essential for maintaining mitochondrial homeostasis and function. Fusion helps to mix mitochondrial contents, supports DNA repair, and maintains membrane potential and ATP production, aiding in stress response and energy demand. Fission segregates damaged mitochondria for degradation and ensures proper distribution during cell division. The balance between these processes is crucial for mitochondrial health, cellular energy production, and overall homeostasis. Therefore, qPCR was used to measure the expression of mitochondrial fusion-related (mitofusin 1 (*Mfn1*), *Mfn2*, and optic atrophy 1 (*OPA1*)) and fission-related (dynamin 1-like (*DNM1L*), mitochondrial fission factor (*Mff*), and fission 1 (*Fis1*)) genes under different treatment conditions. TLZ affected the expression of *Mfn1*, *Mfn2*, *OPA1*, *Mff*, and *Fis1*, but not *DNM1L*, thus indicating that mitochondrial homeostasis in necroptotic astrocytes was disrupted. FANCC overexpression significantly promoted the expression of *Mfn2* and *OPA1* while inhibiting the expression of *Mff* and *Fis1*. Only *Mfn2* expression was significantly affected when FANCC was knocked down. These data indicate that FANCC maintained the balance between mitochondrial fusion and fission, with a particularly notable effect on *Mfn2* (Figure 5E-J).

### FANCC promotes PINK1-Parkin-mediated mitophagy

**Figure 6:**
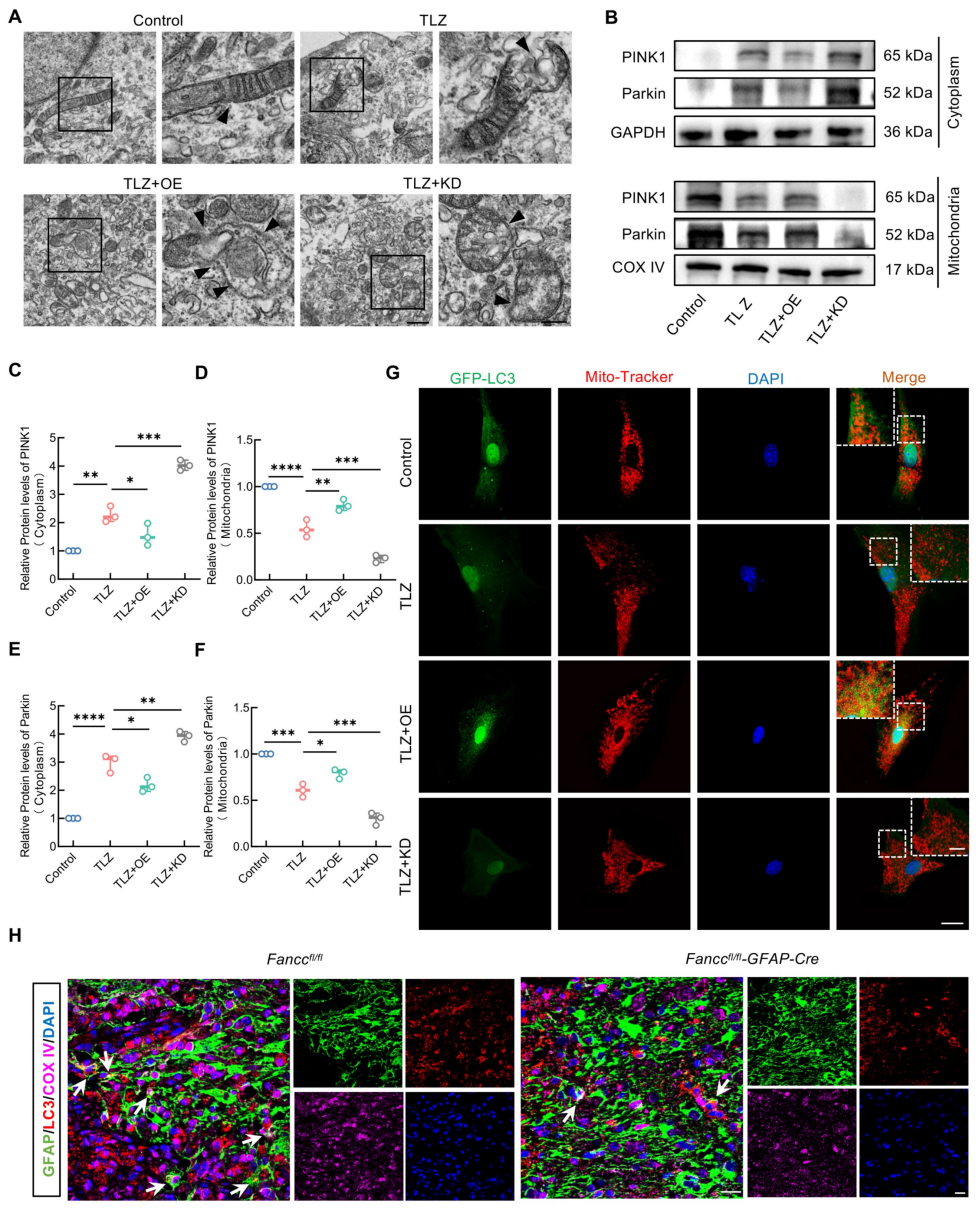
** FANCC promotes PINK1-Parkin-mediated mitophagy.** (**A**) TEM of autophagosome in astrocytes treated with TLZ for 48 h after transfection with FANCC-OE or FANCC-KD; scale bar = 500 nm/250 nm. (**B**) Western blotting of PINK1 and Parkin expression in cytoplasm or mitochondrion of astrocytes treated with TLZ for 48 h after transfection with FANCC-OE or FANCC-KD. (**C-F**) Densitometric analysis of PINK1 and Parkin expression in cytoplasm or mitochondrion. (**G**) Fluorescent localization of autophagosomes and mitochondria using the GFP-LC3 probe and Mito Tracker probe, respectively; scale bar = 20/5 μm. (**H**) Representative IF images of LC3 (red), COX IV (pink), and GFAP (green) in the spinal cord in *Fancc^fl/fl^* and *Fancc^fl/fl^-GFAP-Cre* mice at 7 dpi; scale bar = 20 μm. Data are presented as mean ± SD. Statistical significance was defined as *p < 0.05, **p < 0.01, ***p < 0.001, ****p < 0.0001, and no significance (n.s.).

FANCC precisely inhibited mitochondrial damage and ROS generation in necroptotic astrocytes, helping to maintain mitochondrial homeostasis. However, whether FANCC exerted this effect by regulating mitophagy remained unclear. TEM analysis showed that although necroptotic astrocytes exhibited mitochondrial damage, FANCC overexpression promoted the engulfment of dysfunctional mitochondria and formation of mitophagosomes (Figure 6A). At the molecular level, western blotting indicated that cytoplasmic PINK1 and Parkin failed to relocate to the mitochondria after TLZ treatment, however, FANCC overexpression facilitated the transfer of PINK1 and Parkin to the mitochondria. Conversely, FANCC knockdown inhibited this process (Figure 6B-F). In addition, autophagosome and autolysosome levels were reduced in the cytoplasm of necroptotic astrocytes during TLZ stimulation. However, FANCC overexpression promoted the formation of autophagosomes and autolysosomes in the cytoplasm, which was further inhibited by FANCC knockdown (Figure S7A). Furthermore, mitochondrial and autophagosome fluorescence co-localization revealed that FANCC overexpression promoted autophagosome enrichment in the mitochondria (Figure 6G), indicating that FANCC effectively facilitated mitophagy progression in necroptotic astrocytes. This conclusion was validated in *Fancc^fl/fl^-GFAP-Cre* mice. IF results from injured spinal cords showed that the LC3 expression in astrocytic mitochondria of *Fancc^fl/fl^-GFAP-Cre* mice was significantly lower than that in *Fancc^fl/fl^* mice (Figure 6H). Parkin expression level in astrocytes also decreased accordingly, suggesting that mitochondrial autophagy in astrocytes was impaired in *Fancc^fl/fl^-GFAP-Cre* mice after SCI (Figure S7B-C). Simultaneously, the expression of the autophagy receptor protein p62 was increased in necroptotic astrocytes, suggesting impairment of the autophagy pathway. However, p62 expression decreased significantly after FANCC overexpression, indicating reactivation of the autophagy pathway (Figure S7D-E).

### Astrocyte necroptosis inhibition by FANCC depends on mitophagy activation

The present study demonstrated that FANCC inhibited astrocyte necroptosis after SCI, where FANCC activated mitophagy. To investigate the potential link between these two phenomena, autophagy inhibitor 3-methyladenine (3-MA) was used to block the key step in mitophagy. IF analysis revealed that although FANCC overexpression significantly suppressed the expression of RIPK1, RIPK3, and p-MLKL, addition of 3-MA reversed this effect. Consequently, necroptosis level increased further when mitophagy was inhibited (Figure S8A-F). This finding was validated in a co-culture system. GDNF fluorescence level decreased after mitophagy inhibition (Figure S8G-H). Sholl analysis indicated significantly fewer intersections in neurons containing 3-MA than observed in the FANCC-OE group (Figure S8I-J). Simultaneously, FCM analysis demonstrated increased neuronal apoptosis induced by necroptotic astrocytes in the 3-MA group compared to that in the FANCC overexpression group (Figure S8K-L).

### TBK1 is a potential downstream FANCC target protein in mitophagy pathway activation

**Figure 7:**
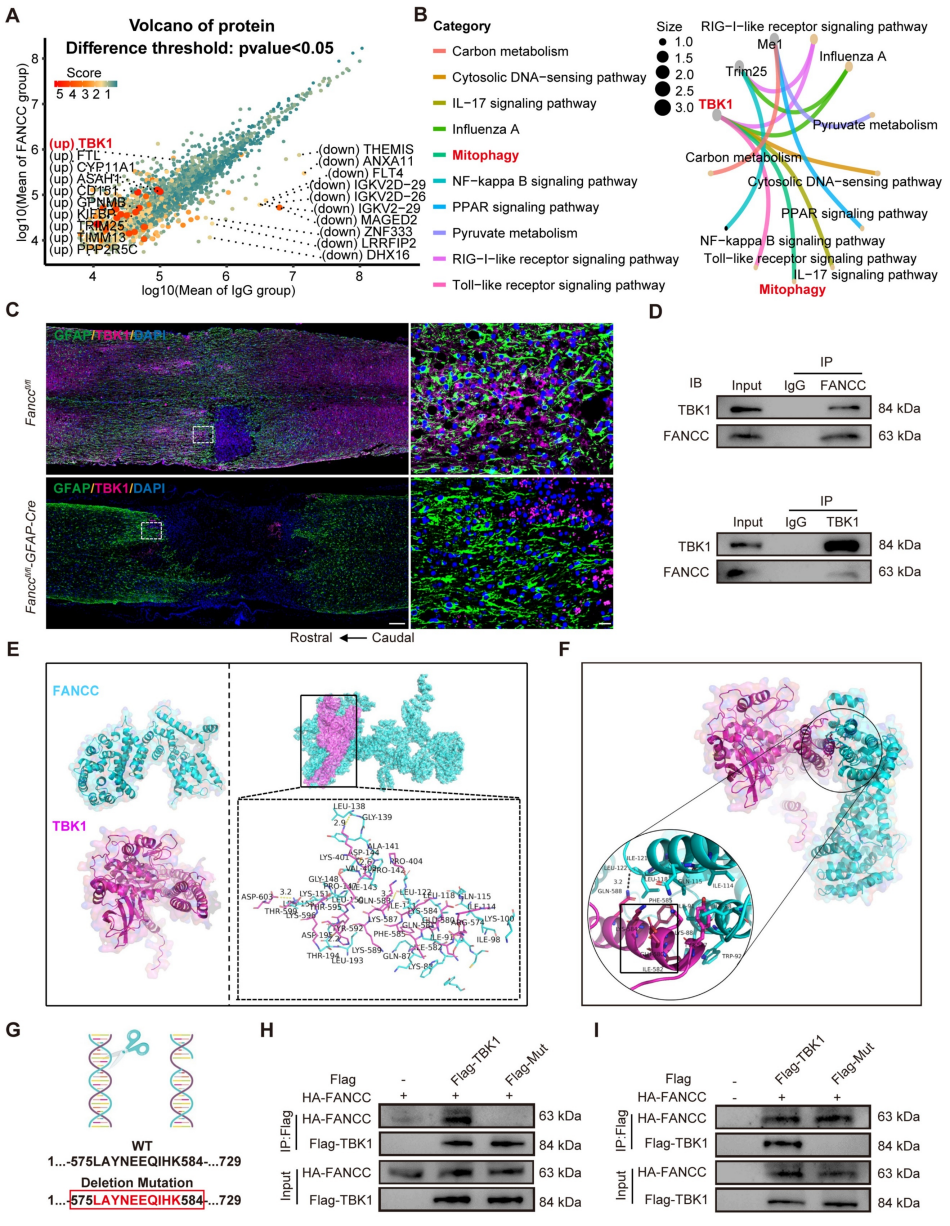
** FANCC activates PINK-Parkin mediated mitophagy through interaction with 575-584 peptide of TBK1.** (**A**) Volcano plot of the protein expression after Co-IP and label-free proteomics. (**B**) KEGG pathway enrichment in mitophagy and TBK1. (**C**) Representative IF images of TBK1 (pink) and GFAP (green) in the spinal cord in *Fancc^fl/fl^* and *Fancc^fl/fl^-GFAP-Cre* mice at 7 dpi; scale bar = 200/20 μm. (**D**) Co-IP assay showing representative protein bands of FANCC and TBK1 in astrocytes. (**E**) Global and detailed views of the FANCC protein docked with TBK1. Cyan surface and cartoon represent FANCC; magenta surface and cartoon represent TBK1. (**F**) Global and local schematic diagrams of the interaction between FANCC and 575-584 peptide of TBK1. (**G**) WT and the sets of designed mutant sequences of 575-584 peptide of TBK1 for validating the predicted binding mode. (**H-I**) HEK293 cells transfected by HA-FANCC and Flag-tagged TBK1 mutant. Co-IP and western blotting of the binding motif between FANCC and TBK1.

Co-IP analysis followed by 5D proteomics evaluation for differential protein analysis was performed to further investigate the molecular mechanism by which FANCC activated mitophagy. A total of 50 upregulated proteins and 148 downregulated proteins were identified (Figure S9A and Table S9). A volcano plot with a cutoff of p<0.05 showed the top 10 upregulated and downregulated proteins (Figure 7A). Lasso regression for penalized dimension reduction analysis was used to determine the downstream proteins regulated by FANCC, identifying downstream proteins with higher relevance to FANCC (Figure S9B and Table S10). Functional enrichment analysis of the selected proteins was then conducted (Figure S9C-E and Table S11-14). These proteins were primarily enriched in selective autophagy in the biological process (Figure S9C). In addition, KEGG pathway enrichment analysis indicated that the proteins were enriched in mitophagy (Figure 7B and Table S14). Only TBK1 played an important role in the mitophagy pathway among these selected proteins, suggesting that TBK1 is involved in the regulation of astrocyte mitophagy by FANCC following SCI (Figure 7B). TBK1 expression was examined further in *Fancc^fl/fl^-GFAP-Cre* mice, and IF revealed that TBK1 expression was significantly lower after SCI than observed in the *Fancc^fl/fl^* group (Figure 7C).

### FANCC activates PINK1-Parkin-mediated mitophagy via interaction with 575-584 peptide of TBK1

The intrinsic connection between FANCC and TBK1 in activating mitophagy in necroptotic astrocytes was investigated. Western blotting showed that FANCC overexpression significantly elevated TBK1 protein levels, whereas FANCC silencing reduced TBK1 expression (Figure S10A-B). The TBK1 inhibitor GSK8612 was introduced to demonstrate that PINK1-Parkin-mediated mitophagy activation by FANCC depended on the presence of TBK1. Although FANCC overexpression enhanced PINK1 and Parkin translocation to the mitochondria, GSK8612 addition significantly blocked these processes, impairing the PINK1-Parkin-mediated mitophagy pathway (Figure S10C-G). In addition, FANCC bound directly to TBK1, which further activated PINK1-Parkin-mediated mitophagy (Figure 7D). Molecular docking analysis to explore the FANCC-TBK1 complex formation revealed a ZDOCK protein-protein docking score of 1,336.793, indicating a stable interaction between FANCC and TBK1. Multiple contact surfaces were observed in the docking results between FANCC and TBK1 (Figure 7E). The 5D proteomics analysis identified the critical TBK1 peptide segment LAYNEEQIHK (575-584) using liquid chromatography-MS/MS (Figure S10H). Further molecular docking studies were conducted to determine whether this peptide segment was crucial for FANCC binding to TBK1. The 580-582 and 584 residues within this segment were binding sites for FANCC and TBK1, suggesting that the LAYNEEQIHK TBK1 peptide was likely a significant binding region for FANCC and TBK1 (Figure 7F). To validate these findings from the computational model, a mutant TBK1 lacking the peptide 575-584 and referred to as Mut was created (Figure 7G). HEK293 cells were transfected with HA-FANCC, Flag-TBK1, and Flag-Mut. Co-IP and western blotting showed that Mut had a markedly reduced binding affinity to FANCC and significantly disrupted the formation of the FANCC-TBK1 protein complex compared to WT TBK1 (Figure 7H-I). These results underscore the crucial role of the TBK1 peptide 575-584 in forming the FANCC-TBK1 complex and activating mitophagy.

### FANCC agonist poliumoside effectively promotes astrocyte mitophagy and inhibits necroptosis following SCI

**Figure 8:**
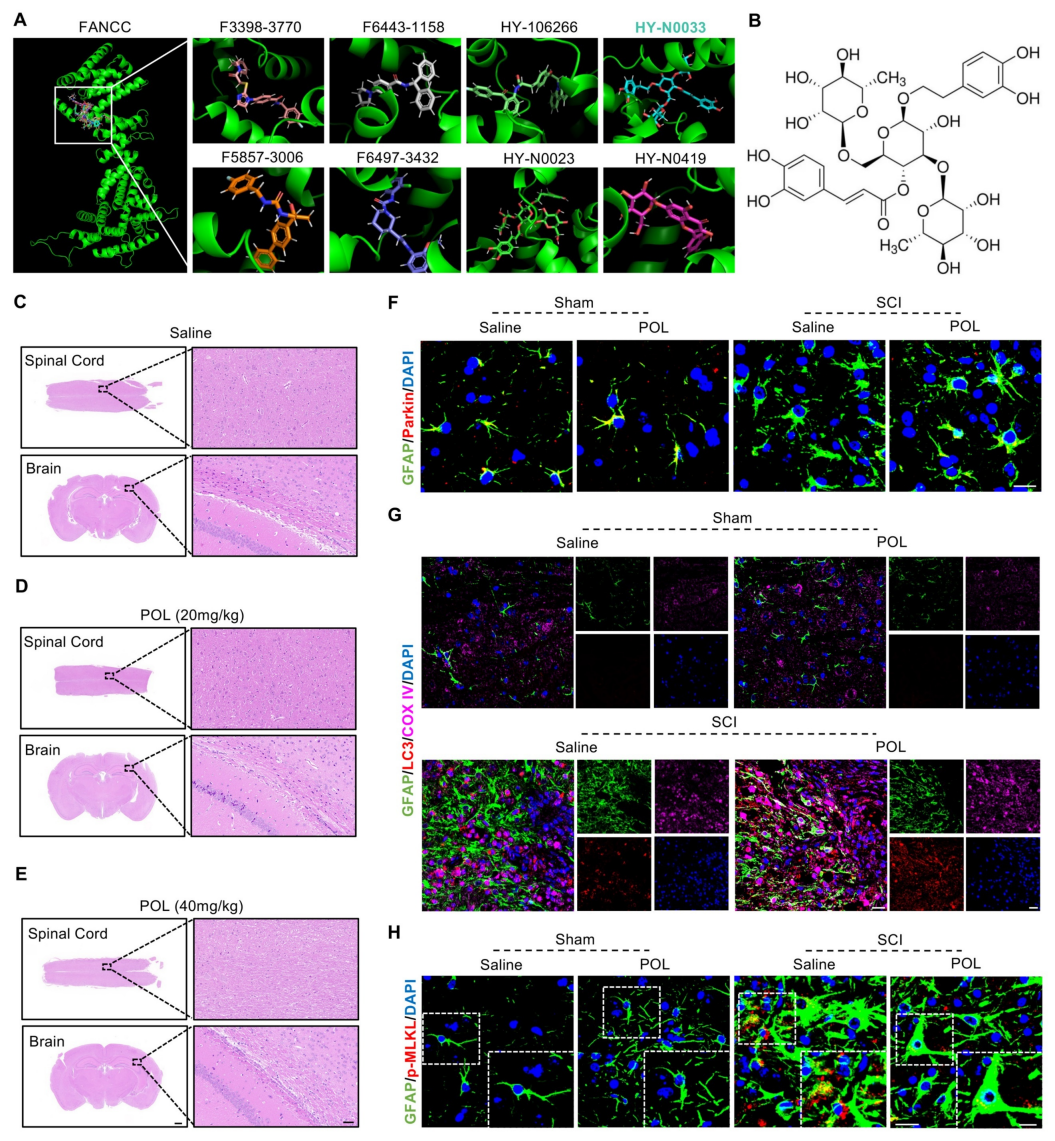
** FANCC agonist poliumoside effectively promotes astrocytic mitophagy and inhibits necroptosis following SCI.** (**A**) Docking complex of FANCC with indicated compounds. (**B**) Chemical structure of poliumoside. (**C-E**) HE staining revealed no apparent damage in the mouse spinal cord and brain following treatment with low and high doses of poliumoside; scale bar = 500/40 μm. (**F**) Representative IF images of Parkin (red) and GFAP (green) in the spinal cord after treatment with poliumoside; scale bar = 10 μm. (**G**) Representative IF images of LC3 (red), COX IV (pink), and GFAP (green) in the spinal cord after treatment with poliumoside; scale bar = 20/20 μm. (**H**) Representative IF images of p-MLKL (red) and GFAP (green) in the spinal cord after treatment with poliumoside; scale bar = 20/10 μm.

On the basis of our aforementioned findings, computer-aided virtual screening was used to identify effective FANCC agonists in order to provide a potential therapeutic strategy for SCI. Molecular docking was performed between FANCC and ~67,200 compounds and screened out the top eight preferred compounds. Poliumoside (HY-N0033) had the highest absolute molecular docking score, indicating the strongest binding affinity to FANCC (Figure 8A-B). Intraperitoneal injections of saline, low-dose (20 mg/kg) and high-dose (40 mg/kg) poliumoside were administered to WT mice to determine the potential toxic side effects of the drug. HE staining indicated that there were no significant changes in the tissue structures of CNS organs (brain and spinal cord) or other organs (heart, liver, spleen, lungs, and kidneys) in the low- and high-dose groups compared to those in the saline group (Figure 8C-E and Figure S11A). Therefore, 40 mg/kg poliumoside was selected for subsequent experiments. The effects of poliumoside treatment on mitophagy and necroptosis in astrocytes of SCI mice were investigated further. IF analysis showed a notable Parkin expression in astrocytes of the local spinal cord in the sham group. However, Parkin expression decreased significantly after SCI. In contrast, SCI mice treated with poliumoside exhibited a marked increase in Parkin expression (Figure 8F). LC3 expression in the mitochondria of astrocytes also significantly increased, indicating that astrocyte mitophagy was activated in the spinal cord of SCI mice treated with poliumoside (Figure 8G). SCI mice treated with poliumoside showed a significant reduction in the expression levels of necroptosis-related proteins p-MLKL, RIPK1, and RIPK3 compared to those in the SCI + saline group, which suggested that poliumoside significantly inhibited astrocyte necroptosis of SCI mice (Figure 8H and Figure S11B-C).

### Poliumoside treatment improves neuropathology and locomotor recovery after SCI

**Figure 9:**
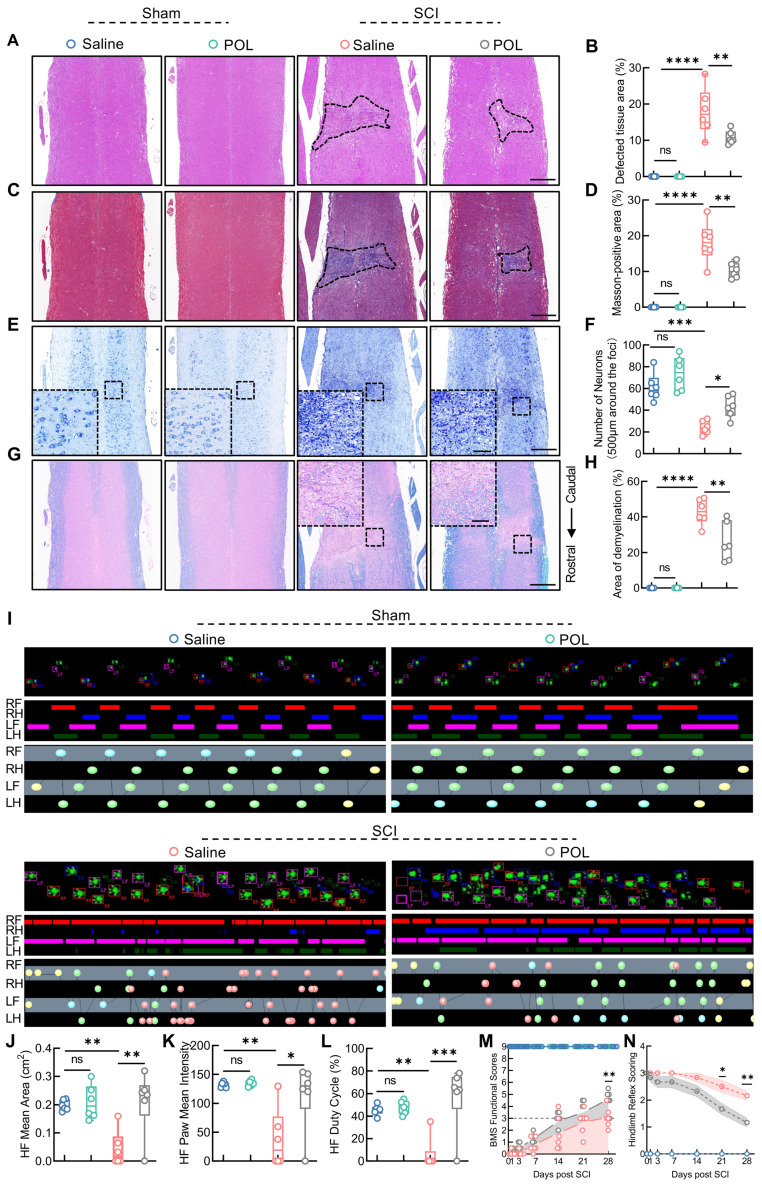
** Poliumoside treatment improved neuropathology and locomotor recovery after SCI.** (**A**) HE staining of the injured spinal cord after treatment with poliumoside (n = 6); scale bar = 400 μm. (**B**) Quantitative analysis of the affected area. (**C**) Masson staining of the injured spinal cord after treatment with poliumoside (n = 6); scale bar = 400 μm. (**D**) Quantitative analysis of the Masson-positive area. (**E**) Nissl staining images of the injured spinal cord after treatment with poliumoside (n = 6); scale bar = 400/80 μm. (**F**) Quantitative analysis of neuronal survival. (**G**) LFB staining of the injured spinal cord after treatment with poliumoside (n = 6); scale bar = 400/80 μm. (**H**) Quantitative analysis of the demyelinated area. (**I**) Representative footprint images were recorded in poliumoside-treated SCI mice (n = 6). The panels are footprints of the hindpaw reflected by green LED light and the colored bands represent standing time of each foot. (**J-L**) The duty cycle, paw mean intensity, and mean area of hindlimb footprints were analyzed by DigiGait software. (**M**) BMS score within 28 dpi in poliumoside-treated SCI mice (n = 6). (**N**) Hindlimb reflex score within 28 dpi in poliumoside-treated SCI mice (n = 6). Data are presented as mean ± SD. Statistical significance was defined as *p < 0.05, **p < 0.01, ***p < 0.001, ****p < 0.0001, and no significance (n.s.).

The neuropathological effects of poliumoside treatment in SCI mice were assessed at 28 dpi using HE, Masson's, Nissl, and LFB staining. Poliumoside treatment significantly reduced the area of spinal cord damage and fibrotic scar formation (Figure 9A-D). It also led to a notable improvement in neuron preservation and alleviated demyelination at the injury site (Figure 9E-H). DigiGait results revealed that the footprint mean area of the hindlimb, paw mean intensity, and duty cycle were all improved significantly after poliumoside administration in SCI mice at 28 dpi compared to those in the SCI+saline group (Figure 9I-L). SCI mice treated with poliumoside showed markedly greater BMS scores than SCI+saline mice starting at 28 dpi (Figure 9M) and higher hindlimb reflex scoring at 21 and 28 dpi (Figure 9N), which collectively demonstrated that poliumoside significantly promoted motor function recovery.

## Discussion

The oxidative storm created by excessive ROS levels wreaks havoc on cellular structures in the aftermath of SCI 51,52. Astrocytes, the guardians of the CNS, succumb to necroptosis within this ROS-rich environment 53. This process begins with the activation of death receptors on the cell surface that recruit and activate RIPK1 and RIPK3, signaling MLKL phosphorylation, which translocates to the plasma membrane, where it oligomerizes and disrupts membrane integrity, leading to catastrophic cell swelling and eventual rupture 54,55. The necroptotic demise of astrocytes is particularly dangerous, as it does not only amplify the oxidative stress but also releases proinflammatory signals that perpetuate a cycle of cellular destruction and inflammation 23,56. This devastating cascade exacerbates the secondary injury, leading to further neuronal dysfunction and impairing the healing process 57. Consequently, necroptotic astrocytes in an ROS-laden milieu profoundly undermine the potential for recovery, leaving a lasting impact on neurological function and underscoring the urgent need for interventions that can break this cycle of oxidative and inflammatory damage following SCI.

The *FANCC* gene is a member of the Fanconi anemia gene family 58,59. Fanconi anemia is a genetically heterogeneous recessive disorder characterized by cellular genetic instability, hypersensitivity to DNA crosslinking agents, increased chromosomal breakage, and defective DNA repair 60,61. The loss of FANCC function leads to a decreased *in vivo* proliferative capacity of multipotent hematopoietic stem cells and plays an indispensable role in Fanconi anemia pathogenesis 62. In addition, FANCC has been shown to be involved in immune processes. For example, Hadjur *et al.* discovered that FANCC-deficient mice are more sensitive to proinflammatory factors, such as interleukin-1β and TNF-α 63. Moreover, FANCC functions in immunity and organellar homeostasis by activating mitophagy 64. Although the role of FANCC in hematological diseases is well-documented, its function in neurological disorders remains largely unexplored. The present study demonstrated for the first time that FANCC was primarily expressed in astrocytes following SCI, and was widely expressed in five identified astrocyte subtypes. Furthermore, we found that astrocytes exhibited distinct cellular states and biological functions at 1d, 4d, and 7d-1m post-injury. Notably, a marked activation of ROS production and neuroinflammation was observed at 7 dpi, which closely correlates with the high expression of FANCC in astrocytes to exert protective function at this time point.

Extensive bioinformatics analyses confirmed that FANCC is a protective gene regulating astrocyte biological functions after SCI. *Fancc^fl/fl^-GFAP-Cre* mice exhibited more severe neural tissue damage and destruction, along with delayed neural and motor function recovery. In addition, astrocytes in *Fancc^fl/fl^-GFAP-Cre* mice showed elevated expression of RIPK1, RIPK3, and p-MLKL, indicating a higher degree of necroptosis. *In vitro*, FANCC overexpression significantly inhibited necroptosis in astrocytes induced by TLZ, while also reducing necroptosis-induced neuronal apoptosis and promoting neuronal survival. Given that neuroinflammation and oxidative stress are key in cavity formation after SCI, the beneficial effects of FANCC in inhibiting astrocytic necroptosis may stem from its protective role in astrocytes, microglia/macrophages, and neurons.

To elucidate the intrinsic mechanisms by which FANCC regulates necroptosis in astrocytes, the study focus was turned back to the astrocyte death process after SCI. As a result, it was found that accumulation of ROS-induced mitochondrial dysfunction was a significant factor leading to necroptosis in astrocytes 65,66. When ROS attacks mitochondrial DNA, it leads to damage in genes, such as uncoupling protein 2 (*UCP2*), voltage-dependent anion channel 1 (*VDAC1*), and peroxiredoxin-3 (*PRDX3*) 67. This disrupts the mitochondrial respiratory chain, collapses MMP, and impairs ATP synthesis, ultimately leading to cell death 68,69. In a modulated ROS environment, damaged mitochondria are efficiently cleared via the PINK1-Parkin-mediated mitophagy pathway, thereby maintaining mitochondrial homeostasis 70,71. However, secondary injury following SCI often results in a ROS storm and uncontrollable mitochondrial damage, hindering mitophagy progression 72. The present research found that FANCC overexpression did not only significantly elevate PINK1-Parkin expression but also stabilized autophagosome and autolysosome formation. This indicated that FANCC markedly activated mitophagy in astrocytes both *in vitro* and *in vivo*, effectively reducing intracellular and mitochondrial ROS accumulation. Rescue experiments further showed that the inhibitory effect of FANCC on necroptosis in astrocytes depended on its mitophagy activation. Thus, it was demonstrated that FANCC inhibited necroptosis following SCI by activating PINK1-Parkin-mediated mitophagy.

Proteomics analysis was used to identify downstream FANCC target proteins in mitophagy activation. TBK1 is a key regulatory kinase that plays a pivotal role in mitophagy orchestration. It is activated in response to mitochondrial damage and translocates to the mitochondria, where it phosphorylates critical mitophagy receptors and adaptors, such as optineurin and p62 73-75. This phosphorylation event enhances the binding affinity of these receptors to the autophagosomal membrane protein LC3, facilitating the engulfment of damaged mitochondria by autophagosomes 76. TBK1 is involved in the stabilization of PINK1 on the outer mitochondrial membrane, promotes Parkin recruitment, and further amplifies this process by phosphorylating ubiquitin chains, creating a strong signal for autophagy receptors to recognize and bind 77. Yuan *et al.* reported that TBK1 suppressed RIPK1-driven apoptosis and inflammation during aging by maintaining mitochondrial quality control and cellular homeostasis 78. Mechanistic studies revealed TBK1 as an essential mediator of FANCC-driven PINK1-Parkin mitophagy activation. Structural mapping identified the 575-584 domain of TBK1 as the critical binding interface for FANCC interaction, and deletion of this region abolished FANCC-TBK1 complex formation and mitophagy activation, thereby inhibiting astrocyte necroptosis. To confirm that FANCC expression can indeed promote neural and motor function recovery after SCI, a specific FANCC agonist poliumoside was identified for SCI treatment. This functional coupling exerted neuroprotective effects through dual mechanisms: 7-day poliumoside treatment triggered robust astrocytic mitophagy while suppressing necroptosis markers. Correspondingly, poliumoside-treated SCI mice demonstrated accelerated functional recovery and reduced lesion volume. However, the described drug treatment in the present study still had some limitations. Although poliumoside, identified as a therapeutic agent in our study, demonstrated efficacy in attenuating pathological cascades following SCI, its non-cell type-specific activity limits precise delineation of astrocytic FANCC's mechanistic role in SCI. This constraint originates from inherent limitations in current computer-aided virtual screening technologies for drug discovery. Consequently, integrating comprehensive pharmacokinetic analyses into virtual screening-based identification of FANCC-specific agonists could optimize the precision and efficiency of this process. Moreover, whether the molecular mechanisms underlying the effects of the poliumoside treatment aligned with our previous research was not further explored. In addition, the recombinant proteins offer advantages, such as reduced off-target effects. Therefore, strategies based on recombinant FANCC protein can be designed. This approach can further boost mitochondrial activity in astrocytes and offer new therapeutic possibilities for SCI improvement.

In conclusion, the present research established a link between the FANCC-TBK1 protein complex and astrocyte mitophagy activation, which inhibited necroptosis following SCI development. Mechanistically, FANCC overexpression facilitated the interaction with TBK1 protein, leading to the activation of the PINK1-Parkin-mediated mitophagy pathway in astrocytes and thereby inhibiting astrocyte necroptosis and SCI progression. The agonist poliumoside maintained mitochondrial homeostasis, and inhibited astrocyte necroptosis while reversing neural tissue damage and promoting locomotor functional recovery after SCI. The study findings underscored the neuroprotective role of FANCC in regulating mitochondrial homeostasis and necroptosis in astrocytes, offering valuable insights for developing new therapeutic strategies for SCI. Future studies should explore the long-term effects of FANCC activation on neuroregeneration and functional recovery after SCI. Additionally, further preclinical investigations are needed to evaluate the therapeutic efficacy and safety of poliumoside or other FANCC-targeting agents in SCI treatment.

## Supplementary Material

Supplementary figures and tables.

## Abbreviations

3-MA: 3-methyladenine
BMS: Basso Mouse Scale
cKO: conditional knockout
Co-IP: co-immunoprecipitation
dpi: days post-injury
FANCC: Fanconi anemia complementation group C
FC: fold change
GEO: Gene Expression Omnibus
HE: hematoxylin and eosin
KEGG: Kyoto Encyclopedia of Genes and Genomes
LC3: light chain 3
LFB: Luxol Fast Blue
LPS: lipopolysaccharide
MLKL: mixed lineage kinase domain-like protein
MMP: mitochondrial membrane potential
PCR: polymerase chain reaction
PINK1: PTEN-induced kinase 1
RIPK: receptor-interacting protein kinase
RNA-Seq: RNA sequencing
ROS: reactive oxygen species
SCI: spinal cord injury
SD: standard deviation
TBK1: TANK-binding kinase 1
TNF-α: tumor necrosis factor-α
TOM20: translocase of outer mitochondrial membrane 20
WT: wild-type
