# Supplementary material for: Activation of FANCC attenuates mitochondrial ROS-driven necroptosis by targeting TBK1-dependent mitophagy in astrocytes after spinal cord injury
Source: Theranostics. 2025 Mar 18;15(9):4188–211. doi: 10.7150/thno.109071 (PMC11980668; doi:10.7150/thno.109071)
Supplement: Supplementary file 1 — Supplementary figures and tables. [file thnov15p4188s1.zip › Supplementary materials/Supplementary Figures.pdf]

# Supplementary Figures

## Figure S1

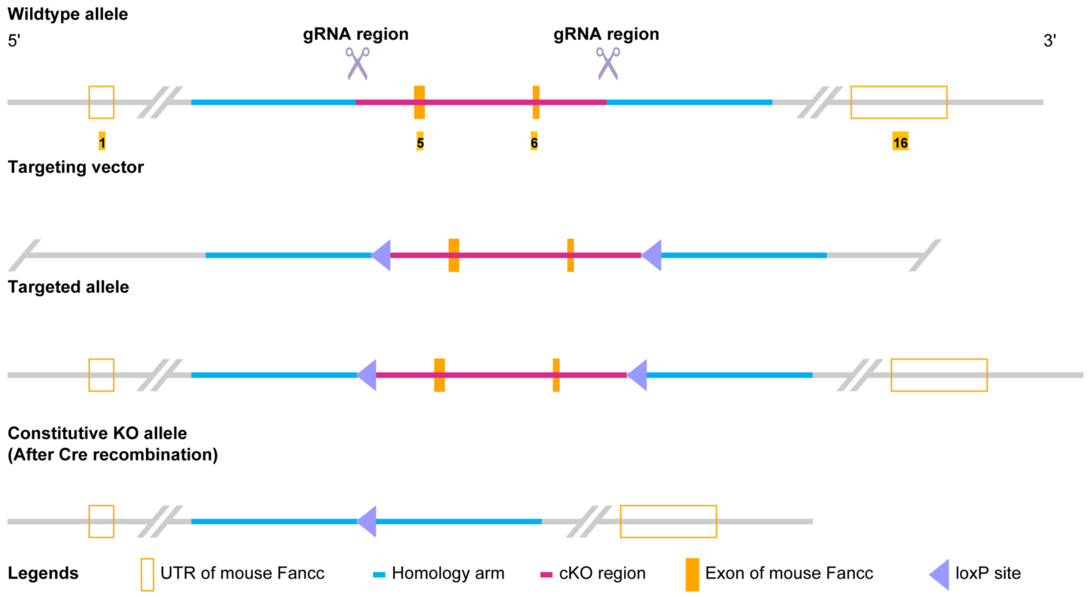


## Figure S1

Targeting strategy for the FANCC cKO mice.

# Figure S2

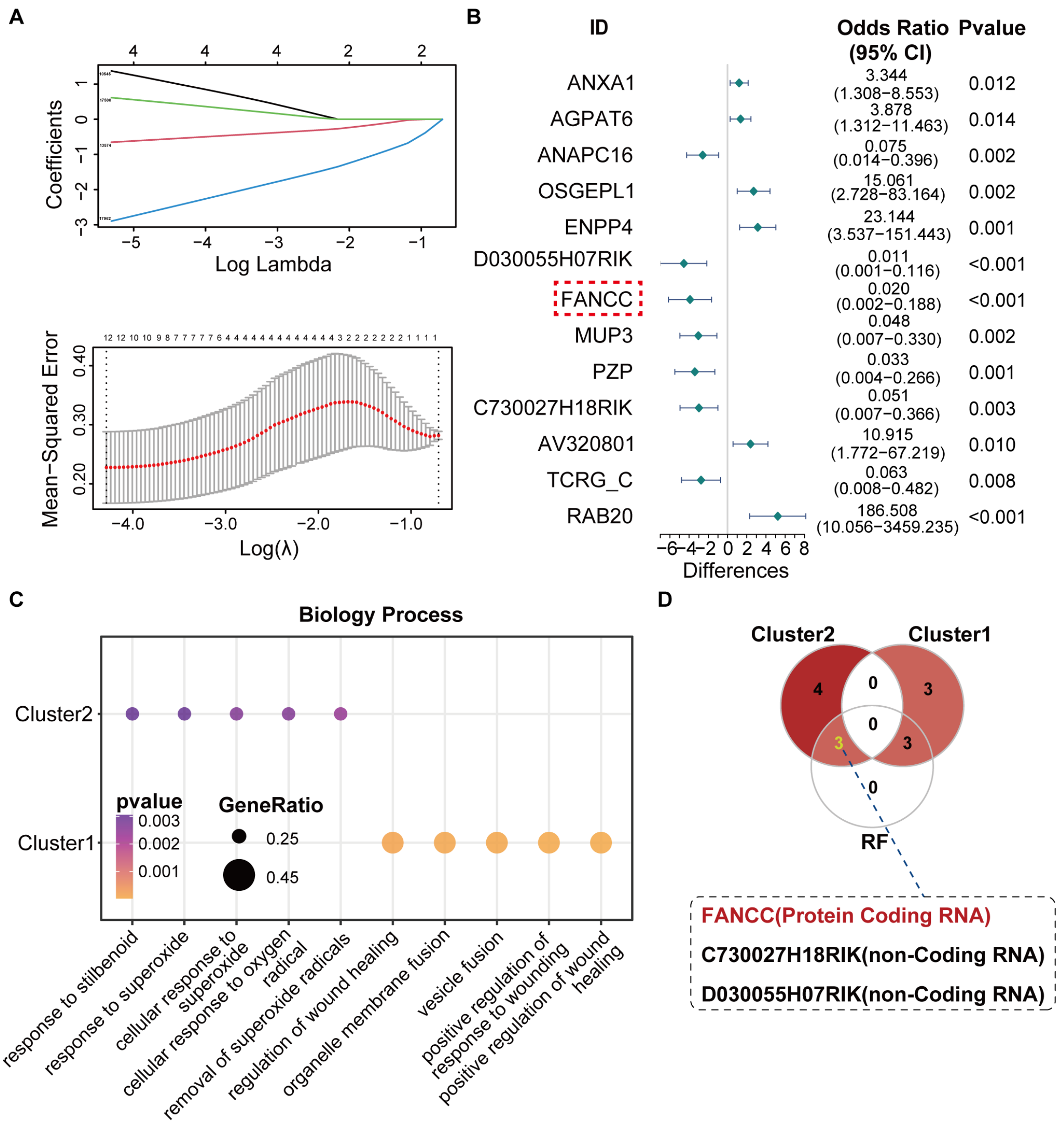


# Figure S2

(**A**) LASSO regression penalized dimension reduction analysis to identify genes closely associated with SCI. (**B**) Logistic regression risk model identified 13 genes associated with SCI. (**C**) Enrichment analysis of genes in Clusters 1 and 2. (**D**) Co-expression analysis between Clusters 1 and 2, according to a random forest model.

## Figure S3

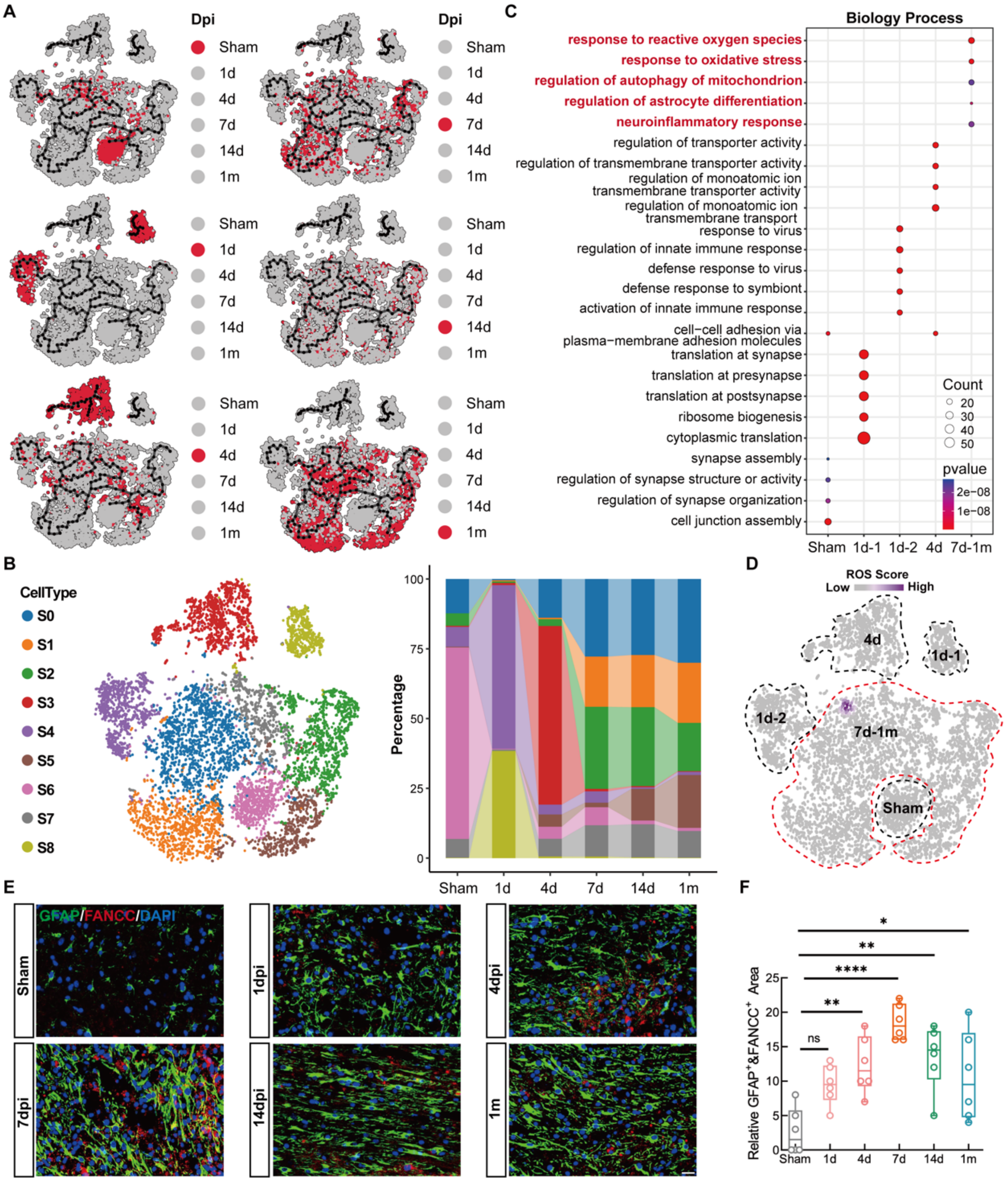


## Figure S3

(**A**) Differentiation trajectories of astrocytes identified at different time points. Red indicates cells detected at each respective time point. (**B**) Proportions of each astrocyte subtype at different time points. (**C**) Bubble plot of the top five biological processes associated with different astrocyte states. The x-axis represents astrocyte states, the y-axis represents GO terms, dot size indicates the number of genes, and dot color

represents the p-value. (**D**) Expression of the ROS Score across five astrocyte states, calculated using a weighted kernel density algorithm. Purple represents the density intensity of the ROS Score. (**E**) Fluorescent expression changes of FANCC in astrocytes of spinal cord within 1m after injury; scale bar = 20 μm. (**F**) Quantitative analysis of FANCC-positive and GFAP-positive area within 1m after injury. Data are presented as mean ± SD. Statistical significance was defined as *$p < 0.05$, **$p < 0.01$, ***$p < 0.001$, ****$p < 0.0001$, and no significance (n.s.).

## Figure S4

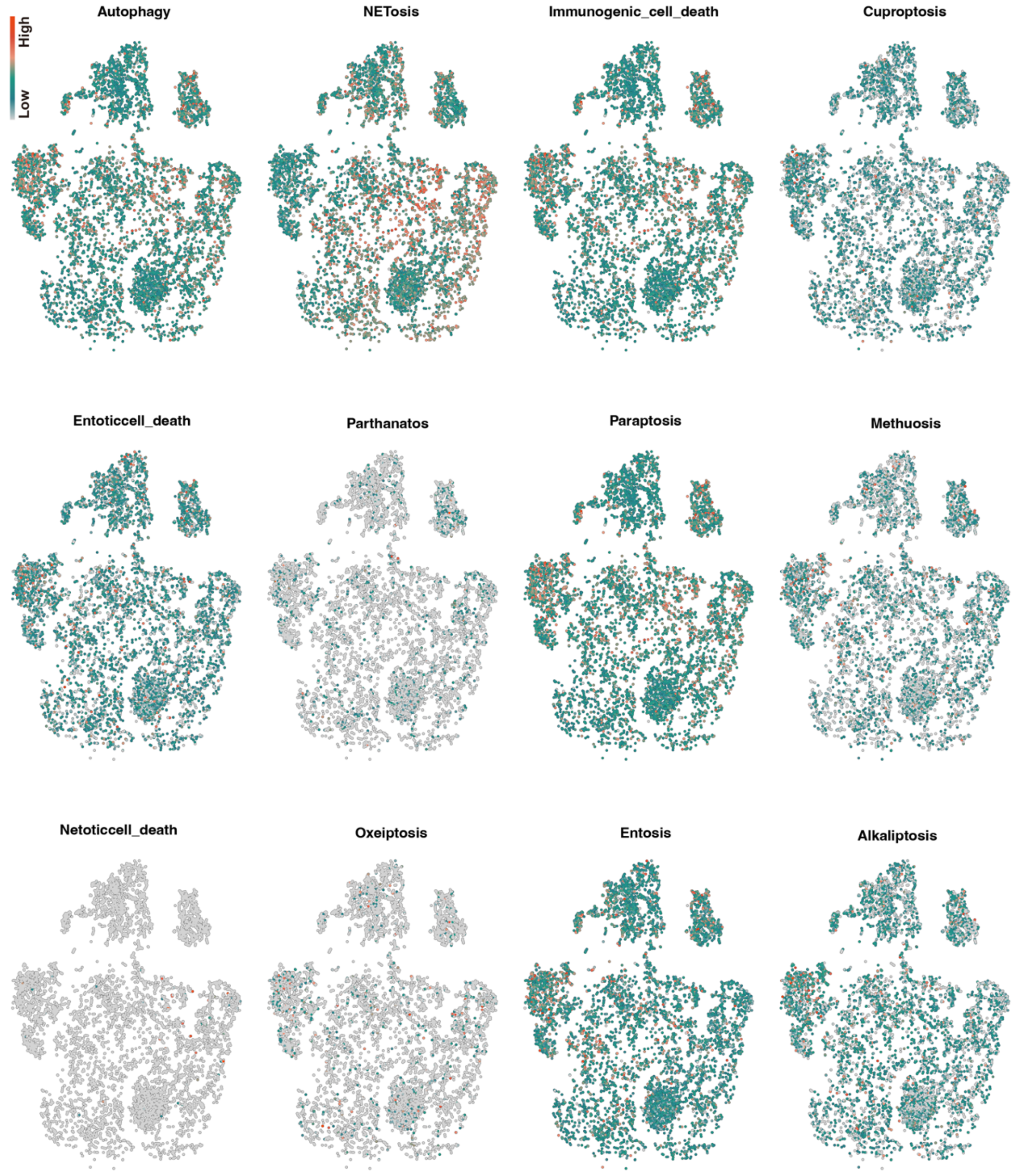


## Figure S4

Scores of 12 cell death phenotypes calculated using the weighted nuclear density algorithm.

## Figure S5

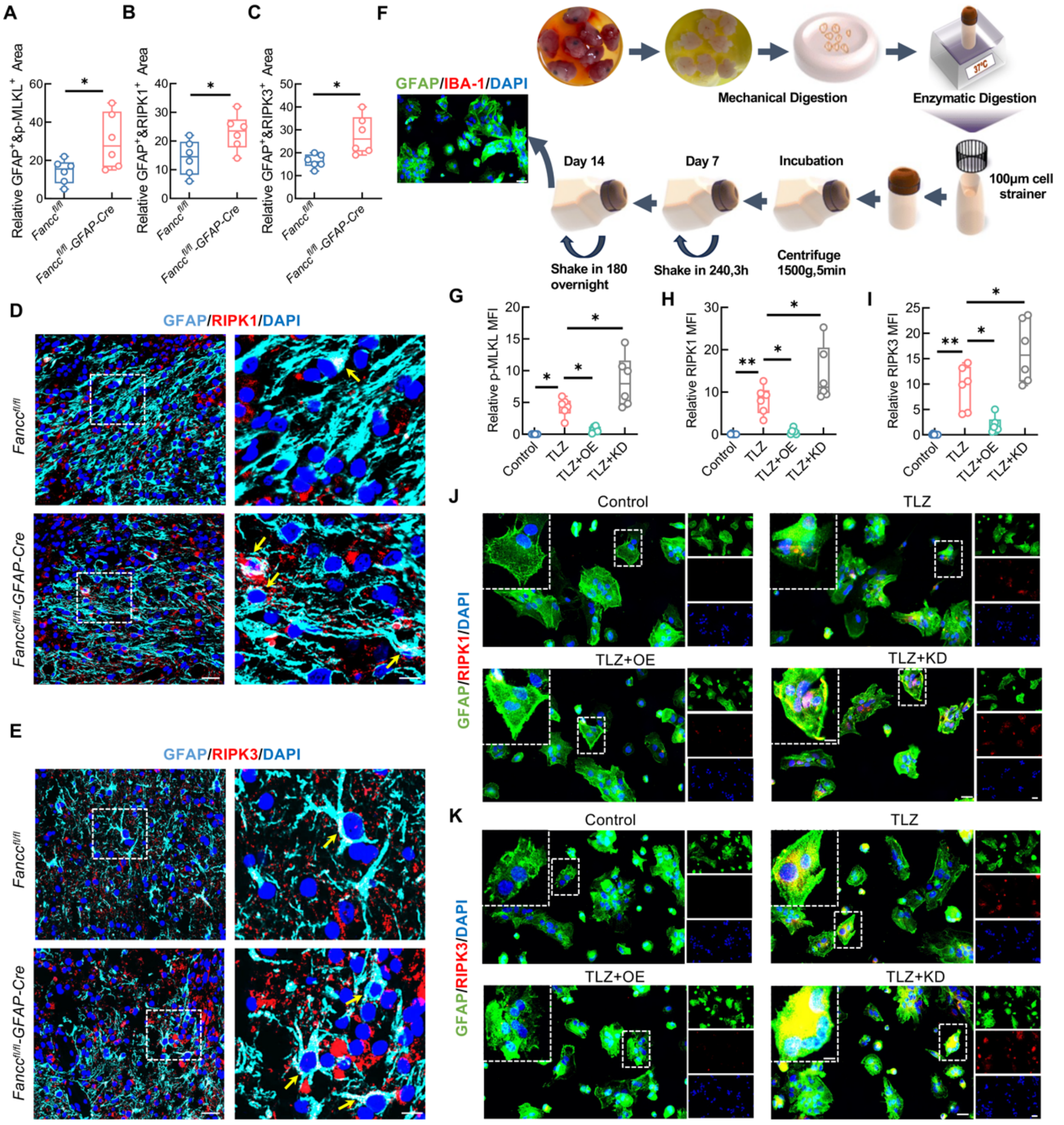


## Figure S5

(**A**) Quantitative analysis of p-MLKL-positive and GFAP-positive area in *Fancc$^{fl/fl}$* and *Fancc$^{fl/fl}$-GFAP-Cre* mice at 7 dpi. (**B**) Quantitative analysis of RIPK1-positive and GFAP-positive area in *Fancc$^{fl/fl}$* and *Fancc$^{fl/fl}$-GFAP-Cre* mice at 7 dpi. (**C**) Quantitative analysis of RIPK3-positive and GFAP-positive area in *Fancc$^{fl/fl}$* and *Fancc$^{fl/fl}$-GFAP-Cre* mice at 7 dpi. (**D**) Representative IF images of RIPK1 (red) and GFAP (green) in the spinal cord in *Fancc$^{fl/fl}$* and *Fancc$^{fl/fl}$-GFAP-Cre* mice at 7 dpi; scale bar = 25/10 μm. (**E**) Representative IF images of RIPK3 (red) and GFAP (green) in the spinal cord in

*Fancc*$^{fl/fl}$ and *Fancc*$^{fl/fl}$*-GFAP-Cre* mice at 7 dpi; scale bar = 25/10 μm. (**F**) Schematic diagram of the extraction process of primary astrocytes. (**G-I**) Quantitative analysis of p-MLKL, RIPK1 and RIPK3 fluorescence expression in astrocytes treated with TLZ for 48 h after transfection with FANCC-OE or FANCC-KD. (**J**) Representative IF images of RIPK1 (red) and GFAP (green) in astrocytes treated with TLZ for 48 h after transfection with FANCC-OE or FANCC-KD; scale bar = 50/20/50 μm. (**K**) Representative IF images of RIPK3 (red) and GFAP (green) in astrocytes treated with TLZ for 48 h after transfection with FANCC-OE or FANCC-KD; scale bar = 50/20/50 μm. Data are presented as mean ± SD. Statistical significance was defined as *$p < 0.05$, **$p < 0.01$, ***$p < 0.001$, ****$p < 0.0001$, and no significance (n.s.).

## Figure S6

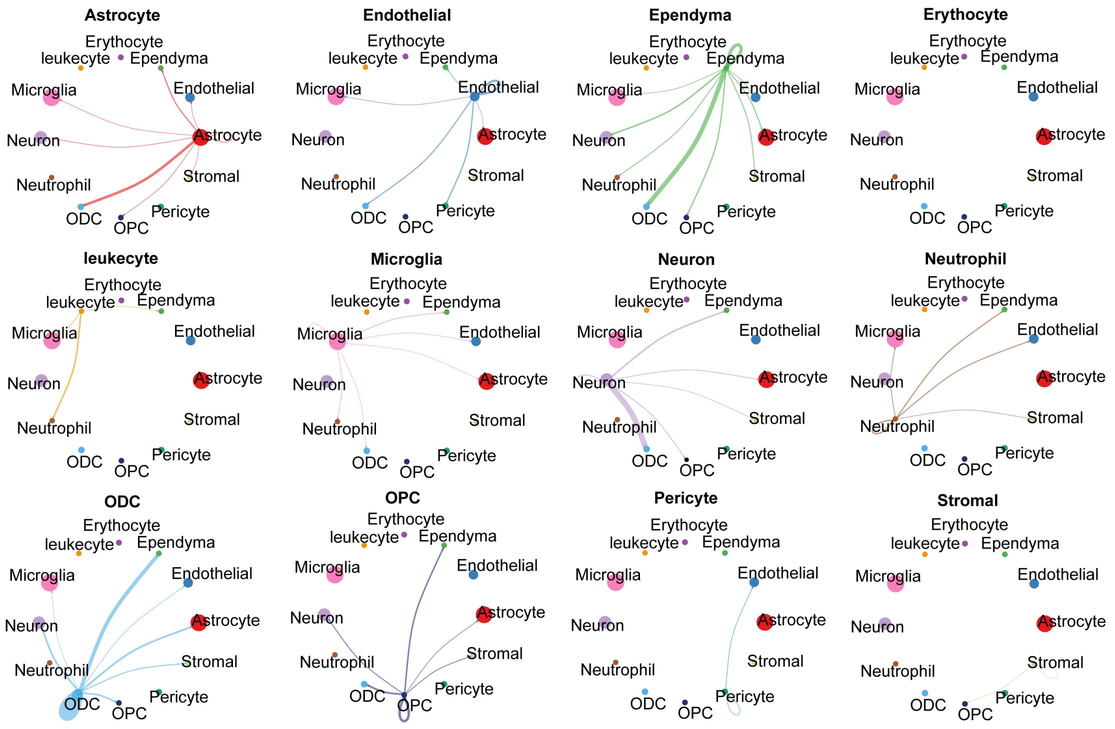


## Figure S6

Interactions between different cell types in the spinal cord after SCI.

**Figure S7**

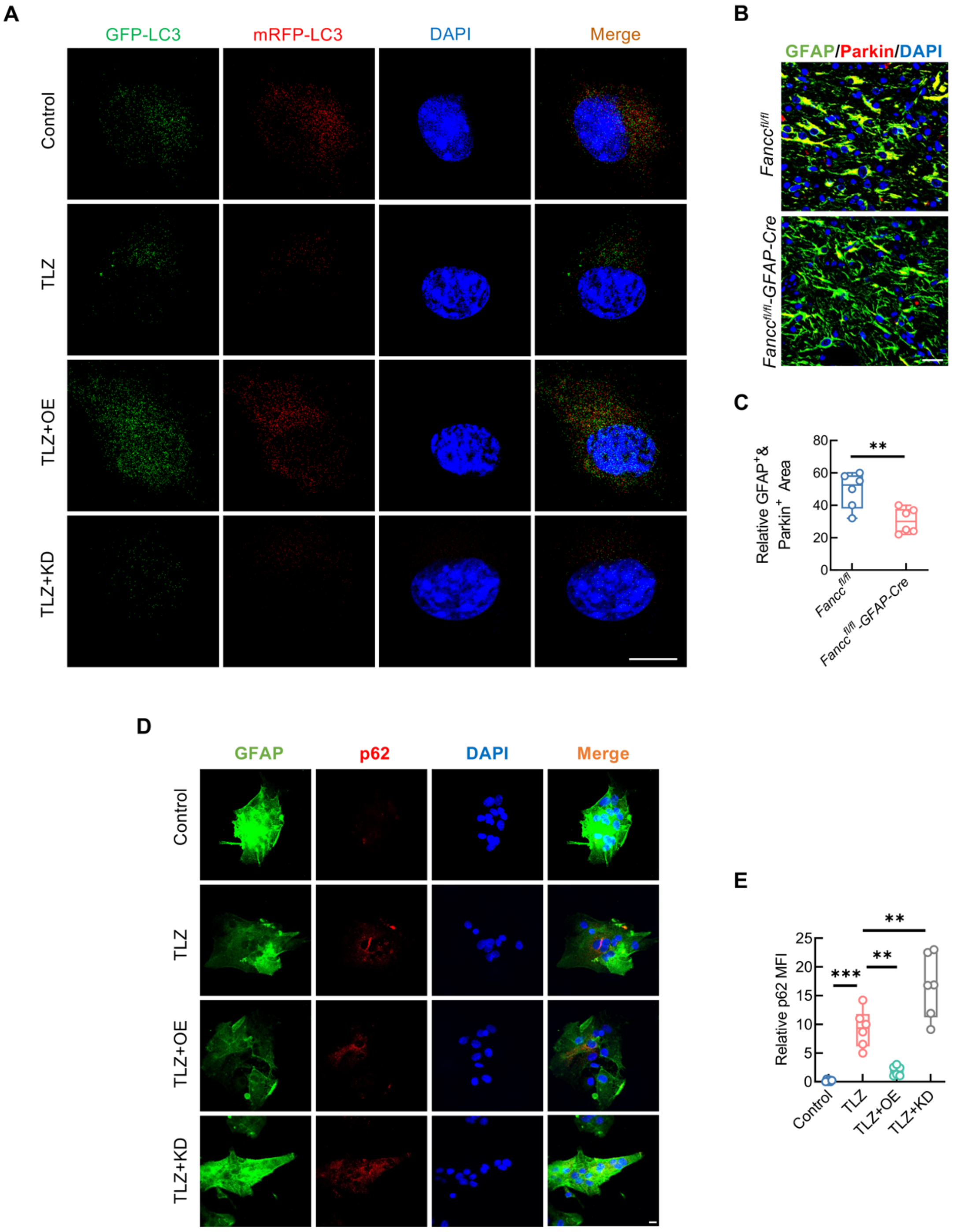


**Figure S7**

(**A**) Fluorescent localization of autophagosomes (yellow) and autolysosomes (red) using the GFP-mRFP-LC3 probe; scale bar = 10 μm. (**B**) Representative IF images of Parkin (red) and GFAP (green) in the spinal cord in *Fancc*$^{fl/fl}$ and *Fancc*$^{fl/fl}$*-GFAP-Cre* mice at 7 dpi; scale bar = 20 μm. (**C**) Quantitative analysis of Parkin-positive and

GFAP-positive area in *Fancc*$^{fl/fl}$ and *Fancc*$^{fl/fl}$*-GFAP-Cre* mice at 7 dpi. (**D**) Representative IF images of p62 (red) and GFAP (green) in astrocytes treated with TLZ for 48 h after transfection with FANCC-OE or FANCC-KD; scale bar = 20 μm. (**E**) Quantitative analysis of p62 fluorescence expression in astrocytes treated with TLZ for 48 h after transfection with FANCC-OE or FANCC-KD. Data are presented as mean ± SD. Statistical significance was defined as *p < 0.05, **p < 0.01, ***p < 0.001, ****p < 0.0001, and no significance (n.s.).

## Figure S8

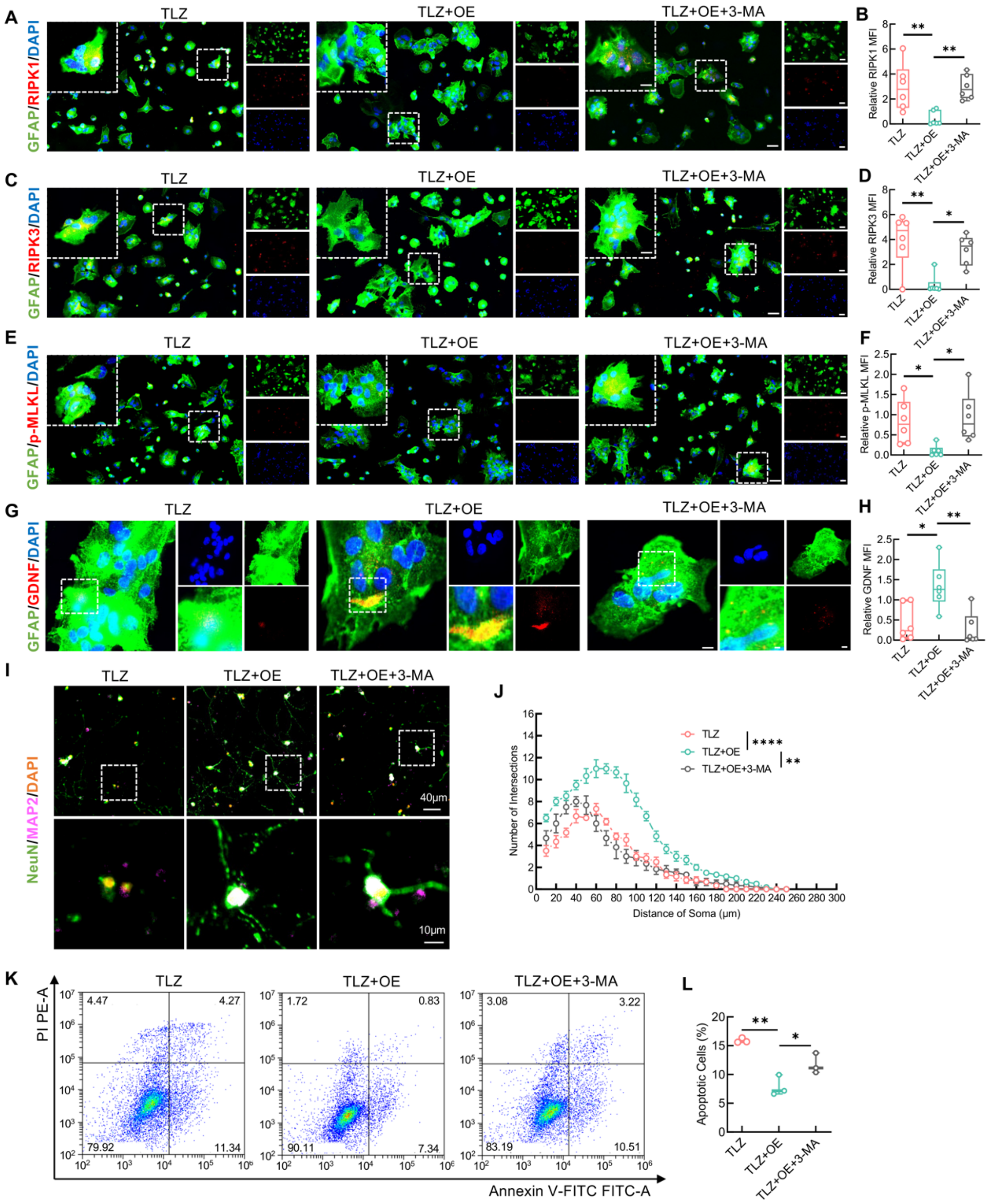

## Figure S8

(**A, C, E, G**) Representative IF images of GFAP (green) and RIPK1, RIPK3, p-MLKL and GDNF (red) in astrocytes treated with TLZ and 5 mM 3-MA after transfection with FANCC-OE; scale bar = 50/20/50 μm. (**B, D, F, H**) Quantitative analysis of RIPK1, RIPK3, p-MLKL and GDNF fluorescence expression in astrocytes treated with TLZ

and 5 mM 3-MA after transfection with FANCC-OE. (**I**) Representative IF images of NeuN (green) and MAP-2 (pink) in neurons; scale bar = 40/10μm. (**J**) Quantification of intersections by Sholl analysis. (**K**) Representative images of FCM results with labeling of PI and annexin-V–FITC in neurons. (**L**) Percentage of apoptotic neurons. Data are presented as mean ± SD. Statistical significance was defined as *$p < 0.05$, **$p < 0.01$, ***$p < 0.001$, ****$p < 0.0001$, and no significance (n.s.).

## Figure S9

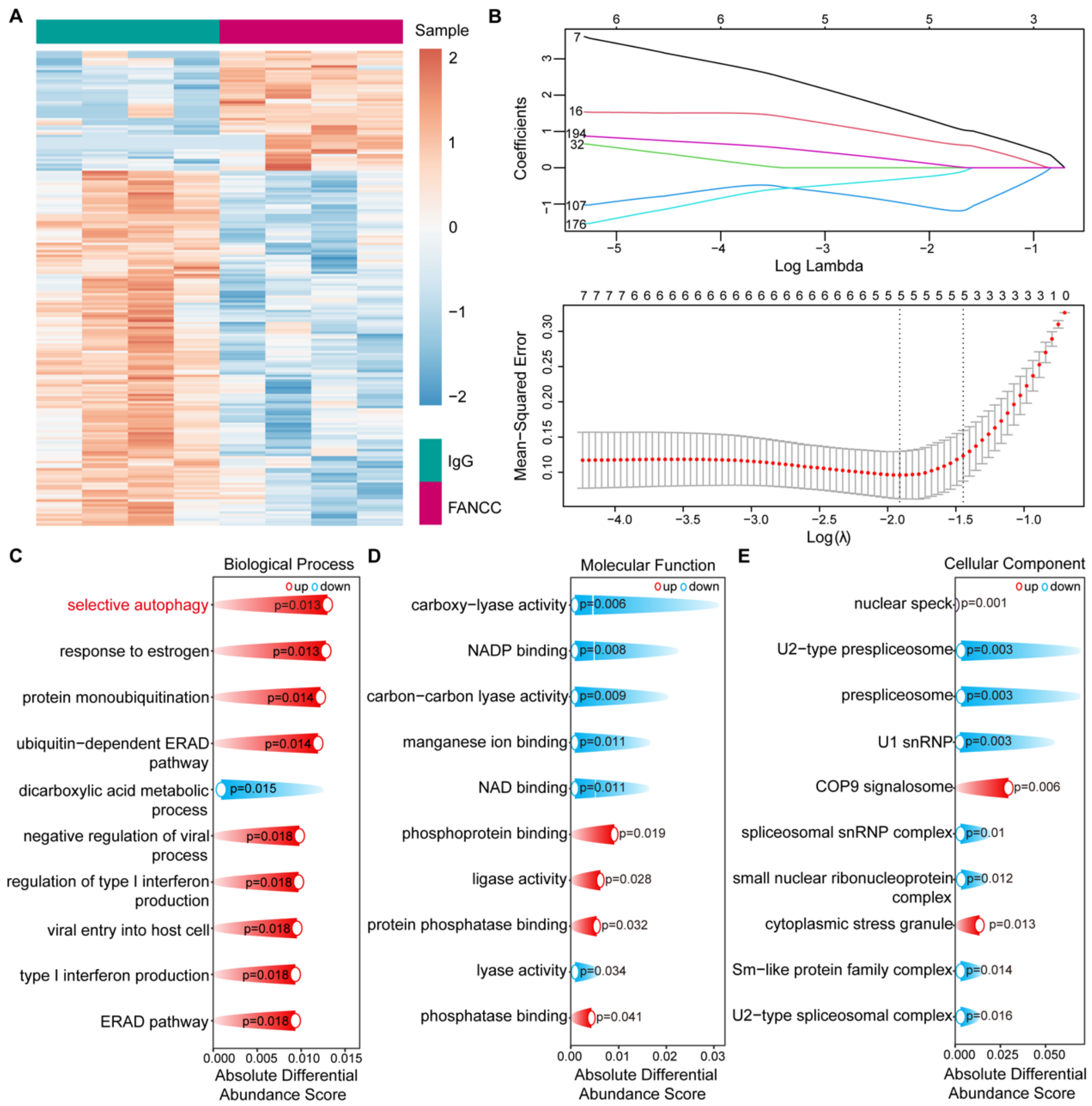


## Figure S9

(**A**) Heatmap of differential protein analysis from 5D proteomics after Co-IP. (**B**) LASSO regression penalized dimension reduction analysis to identify downstream proteins with a higher association with FANCC. (**C-E**) Functional enrichment analysis of the selected proteins. Biological processes were mainly enriched in selective autophagy.

**Figure S10**

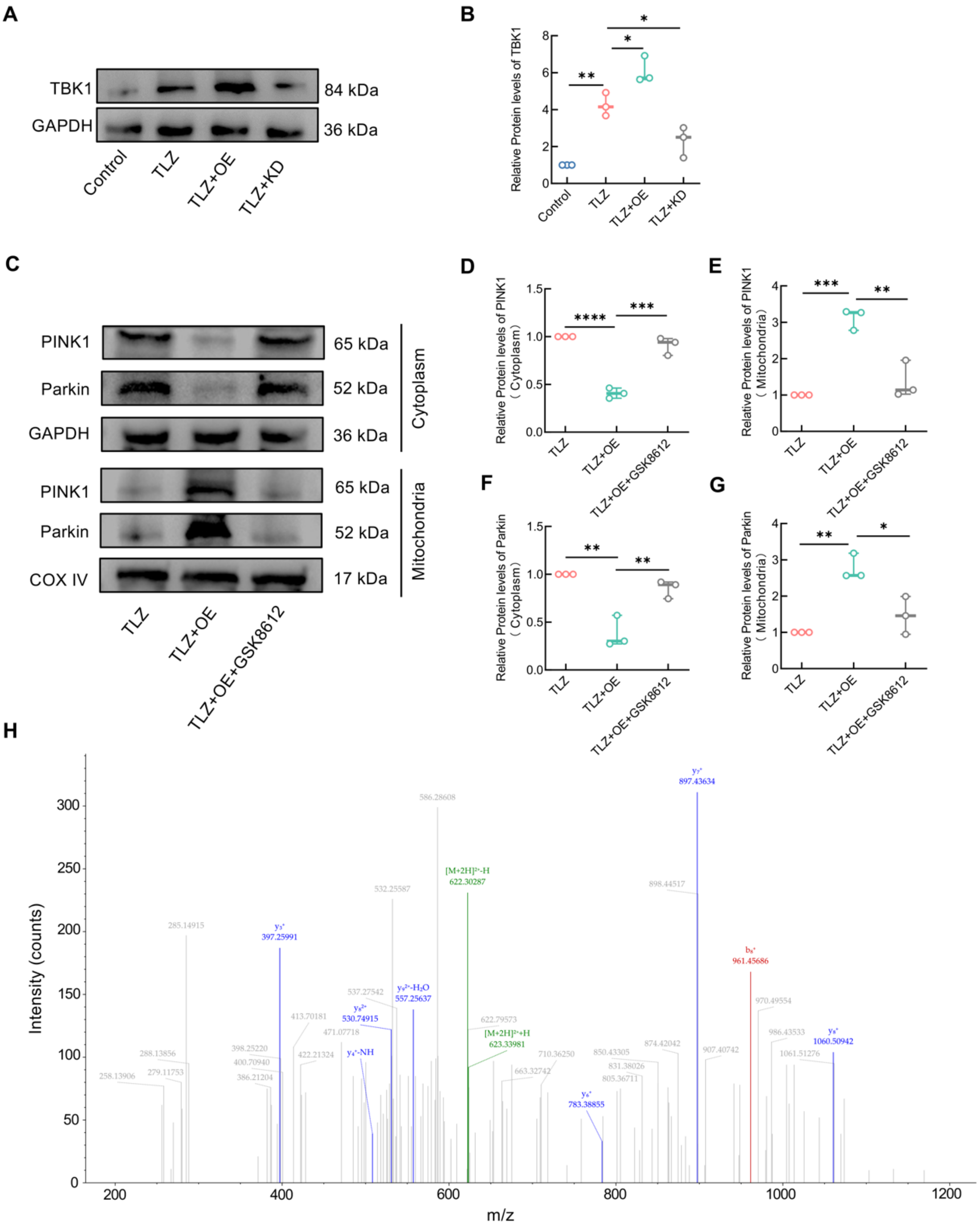


**Figure S10**

(**A**) Western blotting of TBK1 expression in astrocytes treated with TLZ for 48 h after transfection with FANCC-OE or FANCC-KD. (**B**) Densitometric analysis of TBK1 expression. (**C**) Western blotting of PINK1 and Parkin expression in cytoplasm or mitochondria of astrocytes treated with TLZ and 5 μM GSK8612 after transfection with

FANCC-OE. (**D-G**) Densitometric analysis of PINK1 and Parkin expression in cytoplasm or mitochondria. (**H**) MS analysis showed the secondary peak diagram of the TBK1 575-584 peptide. Data are presented as mean ± SD. Statistical significance was defined as *$p < 0.05$, **$p < 0.01$, ***$p < 0.001$, ****$p < 0.0001$, and no significance (n.s.).

## Figure S11

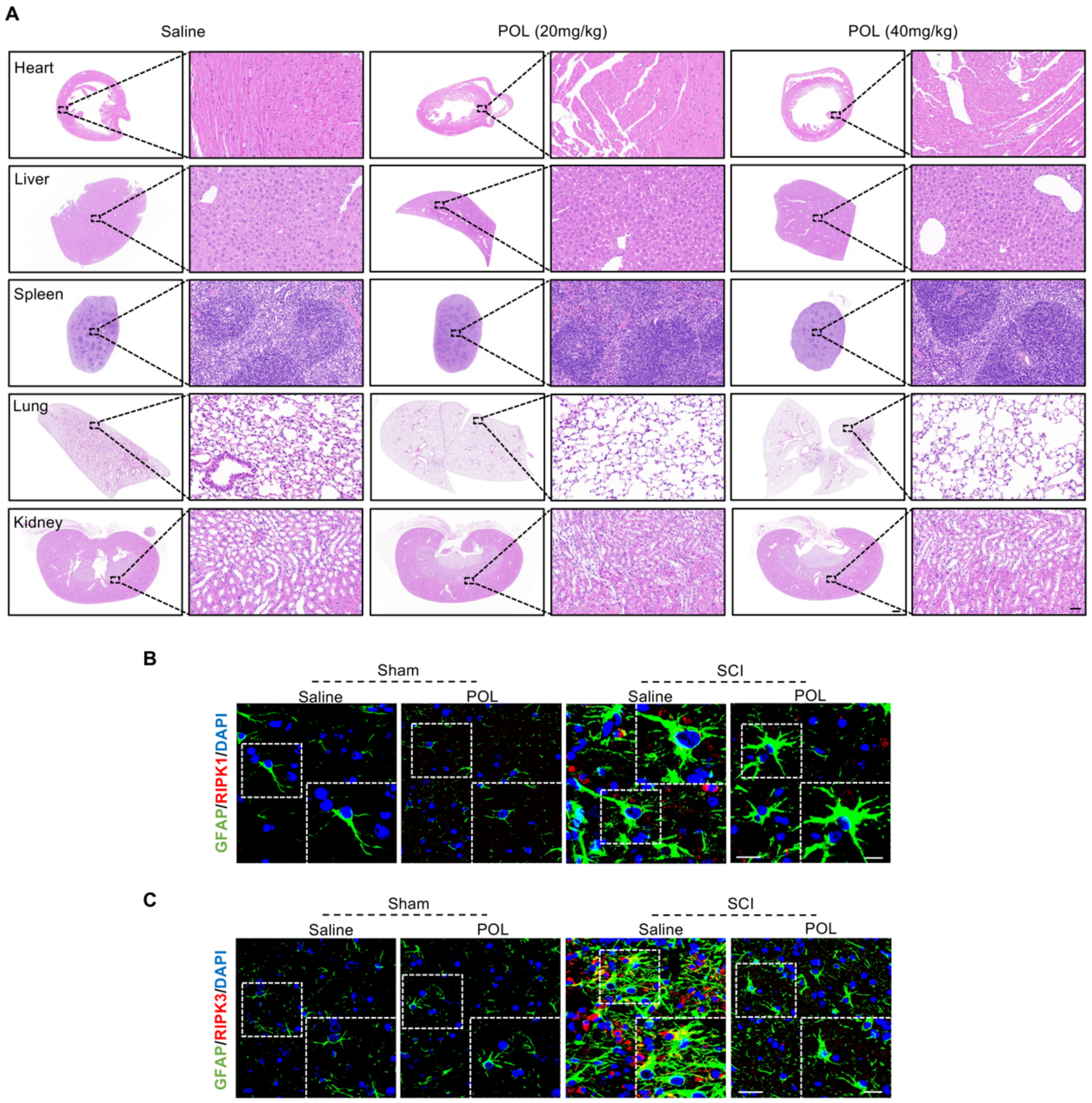


## Figure S11

(**A**) HE staining revealed no apparent damage in the mouse heart, liver, spleen, lungs and kidneys following treatment with low and high doses of poliumoside; scale bar = 500/40 μm. (**B-C**) Representative IF images of RIPK1 or RIPK3 (red) and GFAP (green) in the spinal cord after treatment with poliumoside; scale bar = 20/10 μm.
